# Iron and hepcidin as risk factors in atherosclerosis: what do the genes say?

**DOI:** 10.1186/s12863-015-0246-4

**Published:** 2015-07-11

**Authors:** Tessel E. Galesloot, Luc L. Janss, Stephen Burgess, Lambertus A. L. M. Kiemeney, Martin den Heijer, Jacqueline de Graaf, Suzanne Holewijn, Beben Benyamin, John B. Whitfield, Dorine W. Swinkels, Sita H. Vermeulen

**Affiliations:** Radboud university medical center, Radboud Institute for Health Sciences, Nijmegen, The Netherlands; Department of Molecular Biology and Genetics, Aarhus University, Aarhus, Denmark; Department of Public Health and Primary Care, University of Cambridge, Cambridge, UK; Department of Internal Medicine, VU Medical Centre, Amsterdam, The Netherlands; Department of General Internal Medicine, Division of Vascular Medicine, Radboud university medical center, Nijmegen, The Netherlands; Research Vascular Center Rijnstate, Arnhem, The Netherlands; The University of Queensland, Queensland Brain Institute, St Lucia, Queensland 4072 Australia; QIMR Berghofer Medical Research Institute, Brisbane, Queensland 4029 Australia

**Keywords:** Hepcidin, Iron, Atherosclerosis, Cardiovascular disease, General population, Mendelian randomization

## Abstract

**Background:**

Previous reports suggested a role for iron and hepcidin in atherosclerosis. Here, we evaluated the causality of these associations from a genetic perspective via (i) a Mendelian randomization (MR) approach, (ii) study of association of atherosclerosis-related single nucleotide polymorphisms (SNPs) with iron and hepcidin, and (iii) estimation of genomic correlations between hepcidin, iron and atherosclerosis.

**Results:**

Analyses were performed in a general population sample. Iron parameters (serum iron, serum ferritin, total iron-binding capacity and transferrin saturation), serum hepcidin and genome-wide SNP data were available for *N* = 1,819; non-invasive measurements of atherosclerosis (NIMA), *i.e*., presence of plaque, intima media thickness and ankle-brachial index (ABI), for *N* = 549. For the MR, we used 12 iron-related SNPs that were previously identified in a genome-wide association meta-analysis on iron status, and assessed associations of individual SNPs and quartiles of a multi-SNP score with NIMA. Quartile 4 versus quartile 1 of the multi-SNP score showed directionally consistent associations with the hypothesized direction of effect for all NIMA in women, indicating that increased body iron status is a risk factor for atherosclerosis in women. We observed no single SNP associations that fit the hypothesized directions of effect between iron and NIMA, except for rs651007, associated with decreased ferritin concentration and decreased atherosclerosis risk. Two of six NIMA-related SNPs showed association with the ratio hepcidin/ferritin, suggesting that an increased hepcidin/ferritin ratio increases atherosclerosis risk. Genomic correlations were close to zero, except for hepcidin and ferritin with ABI at rest [−0.27 (SE 0.34) and −0.22 (SE 0.35), respectively] and ABI after exercise [−0.29 (SE 0.34) and −0.30 (0.35), respectively]. The negative sign indicates an increased atherosclerosis risk with increased hepcidin and ferritin concentrations.

**Conclusions:**

Our results suggest a potential causal role for hepcidin and ferritin in atherosclerosis, and may indicate that iron status is causally related to atherosclerosis in women.

**Electronic supplementary material:**

The online version of this article (doi:10.1186/s12863-015-0246-4) contains supplementary material, which is available to authorized users.

## Background

In 1981, the ‘iron hypothesis’ was proposed, stating that iron depletion protects against heart disease [1]. According to this hypothesis, premenopausal women have a lower risk of heart disease compared to men and postmenopausal women due to loss of iron with menstruation. The hypothesis was specified without a mechanism, but it is proposed that high levels of body iron stores promote cardiovascular disease (CVD) by catalyzing low-density lipoprotein (LDL) cholesterol oxidation and thereby atherosclerosis [2, 3]. Since the proposal of the ‘iron hypothesis’, several epidemiological studies have investigated the associations between body iron stores and CVD or (sub)clinical measures of atherosclerosis, but they remain inconclusive [4–12]. The inconsistent results might be explained by the fact that it is not the total body iron load, but the *distribution* of iron among on the one hand parenchymal cells, including the hepatocytes, and on the other hand the macrophages in the spleen as determined by serum hepcidin that drives the association with atherosclerosis and CVD risk. Hepcidin regulates systemic iron homeostasis by controlling the release of iron from i) duodenal enterocytes, involved in dietary iron absorption, ii) macrophages, involved in recycling of iron from senescent erythrocytes, and iii) hepatocytes, involved in iron storage. Increased serum hepcidin concentration leads to a decreased flow of iron into the bloodstream and an increased amount of iron trapped inside the iron-exporting cells, predominantly reticulo-endothelial macrophages [13].

In an extension of the ‘iron hypothesis’ in 2007, hepcidin has been hypothesized to increase CVD risk by slowing or preventing the mobilization of iron from macrophages [14], promoting transformation of these cells into foam cells and ultimately atherosclerosis [3, 14]. In a recent epidemiological study we demonstrated that serum hepcidin and the ratio of hepcidin to ferritin, *i.e.,* hepcidin expression relative to body iron stores, are associated with atherosclerosis in the general population, especially in postmenopausal women [15]. We did not observe associations of the iron parameters, *i.e.,* serum ferritin, serum iron, total-iron binding capacity (TIBC) and transferrin saturation (TS), with atherosclerosis [15]. However, disentangling the specific causal roles of hepcidin and iron parameters in atherosclerosis and CVD in observational population studies is fraught with difficulties due to potential for residual confounding, reverse causation, and the existing phenotypic correlations between iron parameters and hepcidin.

In this study, we aimed to investigate the causal roles of hepcidin, the ratios hepcidin/ferritin and hepcidin/TS, and the iron parameters in atherosclerosis, as measured by non-invasive measurements of atherosclerosis (NIMA), by focusing on their underlying genetics. More specifically, we 1) applied a Mendelian randomization (MR) approach, 2) evaluated associations of genetic determinants of NIMA with hepcidin and iron parameters, and 3) estimated the genomic correlations of hepcidin and the iron parameters with NIMA based on genome-wide chip data.

In the MR approach, genetic determinants of the risk factor(s) of interest, in this case iron status and hepcidin, are used to estimate the causal effect of the risk factor on a disease outcome, in this case NIMA [16]. As genetic variants are randomly distributed in the population, this observational design mimics the randomization in a clinical trial and hence allows for assessment of causality. This is however only valid if three key assumptions hold: 1) the genetic variant must be associated with the exposure, 2) the genetic variant must not directly be associated with the outcome, and 3) the genetic variant must not be associated with any confounding factor.

The second step allowed us to evaluate whether published NIMA-related genetic variants show cross-trait association with hepcidin and the iron parameters. This might indicate presence of pleiotropy, where a single genetic variant affects multiple traits independently. It can also indicate a causal relationship between two correlated traits, where a single genetic variant indirectly affects a second trait (*i.e.,* NIMA) due to a causal association with a first, intermediate trait (*i.e.,* iron and/or hepcidin).

Third, the estimation of genomic correlations allowed us to evaluate the extent to which the same genetic variants, captured via a genome-wide chip, impact on hepcidin or iron parameters and NIMA. Existence of a genomic correlation between two traits can indicate pleiotropy or causality, as for cross-trait associations. A positive genomic correlation indicates that the same genetic variants influence two traits in the same direction, while a negative genomic correlation indicates an opposite direction of effect. The stronger the genomic correlation between two traits, the larger the amount of shared genetic etiology between the traits.

The boost in the identification of genetic variants for complex traits via genome-wide association studies (GWAS) has facilitated the design of MR studies in recent years. For the iron parameters, several GWAS have been published [17–22]. Recently, a large meta-analysis of GWAS on biochemical markers for iron status was completed by the Genetics of Iron Status (GIS) Consortium. The study included 23,986 subjects from eleven population-based studies in the discovery phase and up to 24,986 subjects in the replication phase [23]. This meta-analysis led to the identification of 12 single nucleotide polymorphisms (SNPs) statistically significantly associated with at least one of the iron parameters at a genome-wide level (Additional file 1: Table S1), which we used for the current study in the MR analysis (Additional file 1: Figure S1).

The complex genetic etiology of hepcidin is relatively unexplored. Traglia *et al.* published a GWAS on serum hepcidin in the genetic isolate Val Borbera, in which no statistically significantly associated loci were found [24]. In addition, studies into the SNPs C282Y (rs1800562) in the hereditary hemochromatosis gene (*HFE*) and A736V (rs855791) in the transmembrane serine protease 6 gene (*TMPRSS6*), which have repeatedly been associated with the iron parameters, only associated with the ratios hepcidin/ferritin and hepcidin/TS and not with serum hepcidin [25]. Thus, no genetic determinants of hepcidin are currently available.

There have also been numerous GWAS into genetic determinants of NIMA. Sixteen GWAS were combined in a meta-analysis for common carotid intima media thickness (IMT) and the presence of carotid plaque (total *N* = 42,484), which revealed three (nearest genes *ZHX2*, *APOC1* and *PINX1*) and two (nearest genes *PIK3CG* and *EDNRA*) different SNPs that were statistically significantly associated with IMT and plaque, respectively [26]. A meta-analysis of 21 GWAS for ankle-brachial index (ABI) (total *N* = 58,409) identified one genome-wide significant association with nearest gene *CDKN2B* [27]. We used these six SNPs to study cross-trait associations with iron parameters and hepcidin (Additional file 1: Figure S1 and Table S2).

Here, we studied the roles of hepcidin, the ratios hepcidin/ferritin and hepcidin/TS, and the iron parameters in NIMA by an MR approach, cross-trait associations, and genomic correlations. We used a subsample of 1819 participants aged 42–76 years from the Nijmegen Biomedical Study (NBS), a well-phenotyped sample of the general population.

## Methods

### Study population

Details of the NBS have been described before [28]. Briefly, the NBS is a population-based survey conducted by the Radboud university medical center, Nijmegen, The Netherlands. In 2002, 22,451 age and sex-stratified randomly selected adult inhabitants of Nijmegen, a city located in the eastern part of the Netherlands, received an invitation to fill out a postal questionnaire (QN) including questions about lifestyle, health status, and medical history, and to donate a blood sample for DNA isolation and biochemical studies (NBS-1). A total of 9350 (42 %) persons filled out the QN, of which 6468 (69 %) donated blood samples. In 2005 the second phase of NBS was started (NBS-2), for which all participants of the first phase were re-invited to fill out a second questionnaire. Those who participated in NBS-2 and were aged 50–70 years at that time were asked to also participate in the NIMA (non-invasive measurements of atherosclerosis) substudy performed by the Department of Internal Medicine. For the NBS-2-NIMA study, participants had to fill out an additional QN, donate a fasting blood sample, and undergo anthropometric measurements and non-invasive measurements of atherosclerosis. A total of 1491 subjects participated in NBS-2-NIMA (response 71 %). Approval to conduct the NBS and NBS-2-NIMA study was obtained from the Radboud university medical center Institutional Review Board. All participants gave written informed consent.

Genotype data (Illumina HumanHapCNV370-Duo BeadChip) were available for those 1980 NBS participants that were selected to serve as controls in GWAS [29]. A total of 1819 samples passed quality control [sample yield ≥96 % (after exclusion of intensity-only markers (*n* = 23,573)), Caucasian ancestry ≥90 % (based on Structure analysis), SNP yield ≥96 %]. Measurements of hepcidin and the iron parameters (iron, ferritin, TS and TIBC) were available for all these samples; NIMA for a subset of 549 participants.

### Laboratory methods and clinical measurements

Total serum iron, TIBC, TS and ferritin were measured as described before.^28^ Serum hepcidin was measured with an in-house developed and validated competitive enzyme-linked immunosorbent assay [30, 31]. We used the following NIMA measurements for this study: IMT, presence of plaque and the ankle-brachial index at rest (ABIR) and after exercise (ABIEX). The NIMA and total cholesterol (TC), low-density lipoprotein cholesterol (LDL), high-density lipoprotein cholesterol (HDL), and triglycerides (TGC) were measured as described before [15, 32].

### Selection and measurement of genetic variants

Genome-wide SNP data were used to estimate genomic correlations and were available for 1819 NBS participants, as indicated above. SNP quality control [minor allele frequency (MAF) ≥1 %, and Hardy-Weinberg equilibrium (HWE) *p*-value >10^−6^] resulted in availability of 323,414 SNPs. Density was increased by imputation, which was performed with 1000genomes phase1 integrated version 3 as a reference sample using IMPUTE v2 software [33].

We used in total 18 SNPs for the MR approach and cross-trait associations: 12 iron-related SNPs [23] (Additional file 1: Figure S1 and Table S1) and six NIMA-related SNPs [26, 27] (Additional file 1: Figure S1 and Table S2). Five of these SNPs were directly measured (rs1800562, rs855791, rs744653, rs9990333, and rs6486121) and 13 SNPs were imputed, all of them with a quality of imputation of >0.99 as measured using the SNPtest info measure.

### Statistical analysis

#### SNP associations

**Figure 1:**
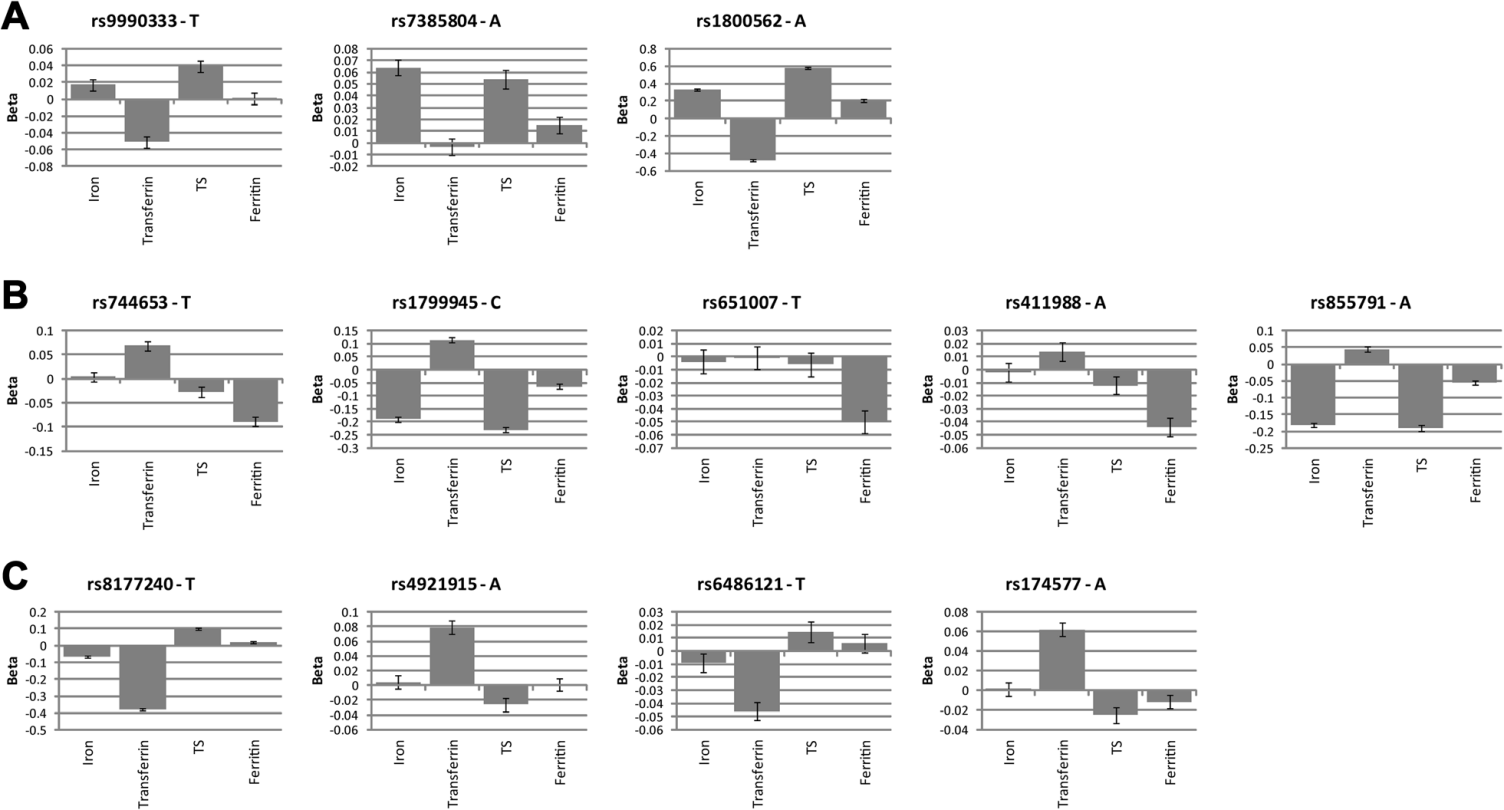
Twelve SNPs identified in meta-GWAS for iron parameters and included in the Mendelian randomization analysis. Betas ± standard error for serum iron, transferrin, transferrin saturation (TS), and ferritin (log) based on the meta-analyses of genome-wide association studies on iron status performed by the Genetics of Iron Status Consortium (discovery and replication combined (*N* = 48,972)). **a** SNPs that are hypothesized to increase the risk of atherosclerosis according to the ‘iron hypothesis’; (**b**) SNPs that are hypothesized to decrease the risk of atherosclerosis according to the ‘iron hypothesis’; (**c**) SNPs for which the hypothesized effect on atherosclerosis risk is unknown

For the MR approach and cross-trait associations, genotype probabilities (P) of the 12 iron-related and 6-NIMA related SNPs were transformed to dosages, *i.e.,* dosage per SNP = P_AA_*0 + P_AB_*1 + P_BB_*2; allele B was the effect allele as presented in Additional file 1: Tables S1 and S2. We also constructed a multi-SNP score that reflects the total number of risk alleles. More specifically, we added the dosages of eight of the 12 iron-related SNPs for which the hypothesized effects on the risk of atherosclerosis according to the ‘iron hypothesis’ were known, thus excluding rs8177240, rs4921915, rs6486121 and rs174577 (see Fig. 1). There is no linkage disequilibrium between these SNPs: six of them are located in different chromosomes, and rs1800562 and rs1799945 (both located in gene *HFE* on chromosome six) show no correlation [r^2^ = 0.001 (based on 1000genomes Phase 3 CEU)]. Reference alleles of rs744653, rs1799945, rs651007, rs411988 and rs855791 were flipped to make sure that all alleles B were the hypothesized risk-increasing alleles according to the ‘iron hypothesis’ (Fig. 1). Hepcidin, the hepcidin ratios and ferritin were log-transformed to normalize their distributions. For these log-transformed variables and for the other iron parameters, standardized residuals adjusted for age, squared age and time of blood sampling were derived. This was done separately for men and women to adjust for gender. Outliers that differed more than four times the SD from the mean were excluded.

Associations between the single SNPs and multi-SNP score and traits were studied using logistic (presence of plaque) and linear (IMT, ABI, hepcidin, hepcidin ratios, iron parameters) regression models. Resulting odds ratios (OR) of logistic regression models for the single-SNP models express the effect of an additional effect allele of the studied SNP on risk of presence of plaque, *i.e.,* AB vs AA and BB vs AB. Resulting betas of linear models express the effect of an additional effect allele of the studied SNP on measures of IMT, ABI, and on standardized residuals of iron, TS, TIBC, log-hepcidin, log-hepcidin/ferritin, log-hepcidin/TS, and log-ferritin. Associations of the multi-SNP score with NIMA were assessed after constructing quartiles (Q1-4) of the score. ORs and betas for the multi-SNP score quartiles were generated for Q2, Q3 and Q4 relative to Q1. We also studied the associations between iron-related SNPs, the multi-SNP score and NIMA with adjustment for the potential confounding factors TC, LDL, HDL and TGC, and stratified by gender.

#### Estimation of genomic correlations and genome-wide SNP explained variances

We estimated genomic correlations (rG) based on only directly measured autosomal SNPs using the software packages GCTA [34] and Bayz [35]. The use of GCTA to estimate rG using genome- or chromosome-wide SNPs for complex traits has been described [36] and applied in other studies before (*e.g.,* [37]). However, recent results of Visscher *et al.* showed that the statistical power to detect statistically significant rG was limited with a sample size of *N* = 1000 [38]. As our sample size for estimation of rG was smaller than 1000, we also estimated rG with a Bayesian multivariate model as implemented in the software package Bayz; this Bayesian analysis handles large sets of traits in a simultaneous analysis, and therefore we expected Bayz to produce more precise estimates compared to GCTA, which is limited to two-trait analysis.

In GCTA, rG are calculated for pairs of traits using the genetic relationship matrix (GRM) and a bivariate restricted maximum likelihood analysis (GREML) [36]. For the estimation of the GRM for pairs of individuals we only retained individuals with a pairwise relationship <0.025 to remove cryptic relatedness from the data, as recommended by Yang *et al.*[39]. This resulted in 423 to 431 individuals for the NIMA and 1456 to 1481 individuals for hepcidin and the iron parameters.

In software package Bayz, Bayesian multivariate models including up to 8 traits simultaneously were used to estimate rG [35, 40]. The genomic model from Janss *et al.* [35] is based on use of an eigenvector decomposition of the GRM in a random regression model; the GRM used is the same one as used in GCTA GREML analyses. The multi-trait implementation makes use of latent variables to model the covariance [40]. To determine the required number of latent variables, the Bayesian Deviance Information Criterion (DIC) [41] was computed. The Bayesian model uses uniform priors for variances to express a-priori ignorance about the model parameters. From the Bayesian model, the marginal posterior means for rG are reported. The marginal posterior standard deviation expresses uncertainty on the estimate, and, with uninformative prior distributions, should be similar to the frequentist SE. For our study, four different multivariate models were constructed to prevent simultaneous inclusion of highly dependent and correlated variables. Thus, hepcidin and ferritin were not included in the same model as the ratio hepcidin/ferritin, as also holds for serum iron and TIBC with TS, *i.e*., the ratio of serum iron over TIBC. Model 1 included hepcidin, ferritin, iron, TIBC, presence of plaque, IMT, ABIR and ABIEX and was used to determine rG for all included variables. Model 2 included the ratio hepcidin/ferritin, iron, TIBC, presence of plaque, IMT, ABIR and ABIEX, model 3 included the ratio hepcidin/TS, ferritin, presence of plaque, IMT, ABIR and ABIEX, and model 4 included hepcidin, ferritin, TS, presence of plaque, IMT, ABIR and ABIEX; these latter three models were used to estimate rG for the ratio hepcidin/ferritin, the ratio hepcidin/TS, and TS, respectively.

Prior to GCTA and Bayz analyses, the following steps were taken: 1) pairwise LD pruning to remove highly correlated SNPs (window size 100, step 5 and r^2^ 0.98) using the software package PLINK v1.07 [42], resulting in 297,574 SNPs for analysis; 2) log-transformation of the variables hepcidin, ferritin and the ratios hepcidin/ferritin and hepcidin/TS to normalize their distributions; 3) transformation of hepcidin, the iron parameters and NIMA to sex-specific residuals using age, squared age and for hepcidin and the iron parameters also time of blood sampling as determinants in regression models; 4) reduction of outliers to mean ± 4SD (maximal number of outliers per trait was six); and 5) standardization of the traits to zero mean and unit variance.

## Results

Characteristics of the subjects included in our study are presented in Table 1. The percentage of males of the total study population was 49 % and median age at inclusion was 63 years. NIMA were available for a subset of 549 participants (49 % male) with a median age of 59 years.

**Table 1:** Characteristics of the study population

Variable†	Total N	Median (P5-P95) or N (%)*
Gender, males	1819	900 (49 %)
Age at inclusion, years	1819	62.9 (42.2–76.0)
Time of blood sampling	1811	
Before 12:00 pm		374 (21 %)
Between 12:00 pm and 5:00 pm		1165 (64 %)
After 5:00 pm		272 (15 %)
Serum hepcidin, nmoles/L	1810	7.5 (0.9–22.5)
Serum ferritin, μg/L	1817	120.7 (17.8–421.4)
Hepcidin/ferritin, μmoles/μg	1808	60.3 (20.9–170.3)
Hepcidin/TS, μmoles/L/%	1791	0.25 (0.04–0.96)
Serum iron, μmoles/L	1800	17.0 (9.0–27.0)
TIBC, μmoles/L	1800	58.0 (46.0–75.0)
TS, %	1800	28.8 (14.1–48.1)
IMT, mm	549	0.85 (0.70–1.05)
ABI at rest	549	1.10 (0.96–1.27)
ABI after exercise	542	1.11 (0.76–1.34)
Presence of plaque	549	232 (42 %)

*ABI* indicates ankle-brachial index, *IMT* intima media thickness, *N* number, *P5* fifth percentile, *P95* 95th percentile, *TIBC* total iron-binding capacity; and *TS* transferrin saturation

*Continuous variables are presented as median (P5-P95). Categorical variables are presented as N (%)

†Hepcidin, ferritin, hepcidin/ferritin, hepcidin/TS, iron, TIBC, TS and time of blood sampling were measured in 2002 and presence of plaque, IMT, and ABI at rest and after exercise were measured between 2005 and 2008

### MR results: associations of iron-related SNPs with IMT, presence of plaque and ABI

Figure 1 visualizes the effects of the SNPs on the iron parameters based on the iron meta-GWAS of the GIS Consortium [23]. The SNPs are classified into their hypothesized effects on the risk of atherosclerosis according to the ‘iron hypothesis’: SNPs that increase serum iron, TS and/or ferritin are hypothesized to increase the risk of atherosclerosis, and vice versa. The hypothesized effect of rs8177240 is classified as unknown, because the T allele of this SNP decreases iron and transferrin and increases TS. The hypothesized effects of rs4921915, rs6486121 and rs174577 are also classified as unknown, because these SNPs are only associated with transferrin.

Results of the multi-SNP score and single SNP association analyses are presented in Table 2. Effects of the multi-SNP score on the NIMA were not directionally consistent with the hypothesized directions of effect. For the single SNP analyses, only the T allele of rs651007, associated with decreased ferritin, showed effects that were consistent with the hypothesized directions of effects for *all* NIMA, with nominal significant effects on IMT [beta −0.021 (95 % CI −0.038; −0.004)] and ABI after exercise [beta 0.034 (95 % CI 0.004; 0.063)]. SNP rs9990333 showed a nominally significant association with presence of plaque that was directionally consistent [OR 1.32 (95 % CI 1.03; 1.68)], but this SNP showed inconsistent, nonsignificant effects on other NIMA. Two other nominally significant effects were found for rs8177240 and rs4921915, but their hypothesized directions of effect were unknown. Adjustment of the single SNP and multi-SNP score associations with NIMA for TC, LDL, HDL and TGC revealed similar results (Additional file 1: Table S3).

**Table 2:** Association of the iron-related SNPs with non-invasive measurements of atherosclerosis

			Presence of plaque			IMT			ABI at rest			ABI after exercise		
SNP – Tested allele	CHR:BP (Build 37)	Freq	H*	OR	95 % CI	H*	Beta	95 % CI	H*	Beta	95 % CI	H*	Beta	95 % CI
Multi-SNP score														
Q1			Ref	Ref	Ref	Ref	Ref	Ref	Ref	Ref	Ref	Ref	Ref	Ref
Q2			>1	1.06	0.66; 1.71	+	−0.024	−0.051; 0.002	-	0.003	−0.022; 0.028	-	−0.024	−0.051; 0.002
Q3			>1	0.82	0.51; 1.34	+	−0.007	−0.034; 0.020	-	0.005	−0.021; 0.030	-	−0.007	−0.034; 0.020
Q4			>1	1.13	0.70; 1.82	+	0.009	−0.018; 0.036	-	0.003	−0.022; 0.028	-	0.009	−0.018; 0.036
rs744653 – T	2:190,378,750	0.86	<1	1.10	0.78; 1.55	-	−0.006	−0.025; 0.014	+	−0.013	−0.031; 0.005	+	−0.019	−0.052; 0.014
rs8177240 – T†	3:133,477,701	0.66	?	1.13	0.87; 1.46	?	−0.010	−0.025; 0.004	?	**0.014**	**0.000; 0.027**	?	**0.030**	**0.005; 0.055**
rs9990333 – T	3:195,827,205	0.47	>1	**1.32**	**1.03; 1.68**	+	0.012	−0.002; 0.025	-	0.006	−0.006; 0.019	-	0.016	−0.007; 0.040
rs1800562 – A	6: 26,093,141	0.06	>1	1.03	0.62; 1.69	+	−0.017	−0.045; 0.011	-	−0.006	−0.032; 0.020	-	−0.005	−0.054; 0.043
rs1799945 – C	6: 26,091,179	0.84	<1	1.19	0.86; 1.64	-	0.011	−0.007; 0.029	+	0.003	−0.014; 0.019	+	−0.006	−0.037; 0.024
rs7385804 – A	7: 100,235,970	0.64	>1	0.92	0.72; 1.18	+	−0.006	−0.020; 0.008	-	0.000	−0.013; 0.013	-	0.018	−0.006; 0.042
rs4921915 – A‡	8: 18,272,466	0.78	?	**1.42**	**1.04; 1.92**	?	−0.006	−0.023; 0.010	?	0.002	−0.014; 0.017	?	0.005	−0.023; 0.034
rs651007 – T	9: 136,153,875	0.20	<1	0.84	0.62; 1.15	-	**−0.021**	**−0.038; 0.004**	+	0.003	−0.013; 0.019	+	**0.034**	**0.004; 0.063**
rs6486121 – T‡	11: 13,355,770	0.64	?	0.94	0.73; 1.20	?	0.012	−0.002; 0.026	?	−0.004	−0.017; 0.009	?	−0.007	−0.031; 0.018
rs174577 – A‡	11: 61,604,814	0.32	?	1.10	0.85; 1.42	?	0.001	−0.013; 0.015	?	0.004	−0.009; 0.017	?	0.015	−0.009; 0.040
rs411988 – A	17: 56,709,034	0.58	<1	0.92	0.73;1.17	-	−0.001	−0.015; 0.012	+	0.004	−0.008; 0.017	+	−0.010	−0.033; 0.013
rs855791 – A	22: 37,462,936	0.46	<1	1.20	0.94; 1.54	-	−0.010	−0.023; 0.004	+	0.005	−0.008; 0.018	+	0.010	−0.013; 0.034

Associations were tested using logistic (presence of plaque) and linear regression (IMT and ABI at rest and after exercise). Resulting odds ratios (OR) of logistic models for the multi-SNP score express the change in odds for presence of plaque relative to Q1. Resulting betas of linear models for the multi-SNP score express the change in IMT or ABI using Q1 as a reference, thus Q2 vs Q1, Q3 vs Q1 and Q4 vs Q1. Resulting ORs of logistic regression models for the single SNP analyses express the effect of each extra tested allele on odds for presence of plaque. Resulting betas of linear models for the single SNP analyses express the effect of each extra tested allele on IMT or ABI. Tested alleles are the same as in the original publication [23]. Nominally significant associations are indicated in bold

*ABI* indicates ankle-brachial index, *CHR:BP* chromosome:base-pair position, *CI* confidence interval, *Freq* frequency of tested allele, *H* hypothesized effect, *IMT* intima media thickness, *OR* odds ratio, *Ref* reference, *Q* quartile, *SNP* single nucleotide polymorphism

*Hypothesized effect on the NIMA according to the ‘iron hypothesis’ (see Fig. 1). Presence of plaque, a higher IMT and a lower ABI indicate presence of atherosclerosis

†This SNP decreases iron and transferrin and increases TS, so the hypothesized effect on atherosclerosis is unknown

‡These SNPs only show association with transferrin, so the hypothesized effect on atherosclerosis is unknown

Stratification by gender revealed no directionally consistent associations of the multi-SNP score with all NIMA in men. However, in women all directions of effect of Q4 compared to Q1 of the score were directionally consistent with the hypothesized direction of effect (Additional file 1: Table S4). This included a nominally significant association for Q4 vs Q1 of the score with IMT [beta 0.033 (95 % CI 0.000;0.067)]. For the single SNP analyses, effects of rs651007 were consistent with the hypothesized directions of effects for *all* NIMA in men and for all NIMA except for ABI at rest in women (Additional file 1: Table S4). The effect of rs651007 on IMT was stronger in women, whereas the effect of rs651007 on presence of plaque and ABI after exercise was stronger in men. In addition to rs651007, rs855791 showed consistent associations with all NIMA in men, whereas rs1799945 and rs7385804 showed consistent associations with all NIMA in women. Effect estimates of the single SNP associations were not systematically higher or lower in men or women. Associations of the gender-specific results remained similar after adjustment for TC, LDL, HDL, and TGC (Additional file 1: Table S5).

### Association of NIMA-related SNPs with the iron parameters and hepcidin

Associations of top SNPs for IMT, plaque and ABI with hepcidin, the ratios hepcidin/ferritin and hepcidin/TS, and the iron parameters are shown in Additional file 1: Table S6. Three nominally significant associations were observed. The IMT-associated SNP rs11781551 showed association with the ratio hepcidin/ferritin, in addition to the ABI-associated SNP rs10757269, and observed directions of effect were consistent with hypothesized directions for both SNPs.

### Genomic correlations

Tables 3 and 4 show the genomic correlations of hepcidin, the ratios hepcidin/ferritin and hepcidin/TS, and the iron parameters with NIMA as obtained by GCTA (Table 3) and Bayz (Table 4). Point estimates of genomic correlations resulting from the two methods were mostly dissimilar. A substantial part of the estimates by GCTA resulted in values of 0, −1 or 1, indicating convergence of the models to extremes, as expected due to our relatively small sample size.

**Table 3:** Genomic correlations (SE) estimated with GCTA

	Presence of plaque	IMT	ABI at rest	ABI after exercise
Hepcidin	0.18 (1.48)	0.11 (1.49)	0.01 (1.29)	0.02 (1.40)
Ferritin	0.15 (1.49)	−1.00 (4.74)	1.00 (38.1)	0.01 (1.45)
Hepcidin/ferritin	−1.00 (4.35)	0.09 (4.35)	−1.00 (>4E5)	0.25 (2.39)
Hepcidin/TS	−1.00 (15.38)	−1.00 (4.87)	0.02 (1.37)	0.01 (1.24)
Iron	0.05 (1.53)	1.00 (4.18)	0.08 (1.05)	−1.00 (38.1)
TIBC	1.00 (17.3)	0.04 (1.16)	1.00 (55.5)	−1.00 (113.7)
TS	0.06 (1.36)	1.00 (147.7)	0.06 (0.96)	1.00 (>1E5)

*ABI* indicates ankle-brachial index, *IMT* intima media thickness, *SE* standard error, *TIBC* total iron-binding capacity; and *TS* transferrin saturation

**Table 4:** Genomic correlations (SE) estimated with Bayz

	Presence of plaque	IMT	ABI at rest	ABI after exercise
Hepcidin*	0.01 (0.27)	−0.01 (0.31)	−0.27 (0.34)	−0.29 (0.34)
Ferritin*	−0.03 (0.28)	0.01 (0.32)	−0.22 (0.35)	−0.30 (0.35)
Hepcidin/ferritin†	0.06 (0.21)	−0.02 (0.24)	−0.10 (0.27)	−0.01 (0.28)
Hepcidin/TS‡	0.12 (0.19)	0.10 (0.21)	−0.07 (0.27)	0.03 (0.29)
Iron*	−0.04 (0.20)	0.06 (0.21)	0.04 (0.25)	0.09 (0.26)
TIBC*	−0.01 (0.14)	0.04 (0.15)	−0.08 (0.16)	−0.07 (0.17)
TS§	−0.01 (0.19)	0.00 (0.19)	0.05 (0.21)	0.04 (0.22)

*ABI* indicates ankle-brachial index, *IMT* intima media thickness, *SE* standard error, *TIBC* total iron-binding capacity, and *TS* transferrin saturation

*Correlations in these rows come from an 8-trait analysis including hepcidin, ferritin, iron, TIBC, presence of plaque, IMT, ABI at rest and ABI after exercise

†Correlations in this row come from a 7-trait analysis including the ratio hepcidin/ferritin, iron, TIBC, presence of plaque, IMT, ABI at rest and ABI after exercise

‡Correlations in this row come from a 6-trait analysis including the ratio hepcidin/TS, ferritin, presence of plaque, IMT, ABI at rest and ABI after exercise

§Correlations in this row come from a 7-trait analysis including hepcidin, ferritin, TS, presence of plaque, IMT, ABI at rest and ABI after exercise

Bayz estimates of genomic correlations with NIMA were very weak (*i.e.,* close to 0), except for the genomic correlation of hepcidin and ferritin with ABI at rest [−0.27 (SE 0.34) and −0.22 (SE 0.35), respectively] and ABI after exercise [−0.29 (SE 0.34) and −0.30 (0.35), respectively].

Evidence for and against a role of hepcidin and the iron parameters in atherosclerosis based on the results of the current study is summarized in Table 5.

**Table 5:** Summary of evidence for and against a role of hepcidin and the iron parameters in atherosclerosis resulting from this study

Trait	Evidence for causal role	Evidence against causal role
Hepcidin	Weak genomic correlations with ABI at rest and ABI after exercise	- No nominally significant associations with all NIMA-related SNPs
		- Genomic correlations with presence of plaque and IMT ~0
Ferritin	- Directionally consistent associations of the multi-SNP score with NIMA in women	- No directionally consistent associations of the multi-SNP score with NIMA in men
	- Directionally consistent associations of rs651007 with NIMA	-No directionally consistent associations of other variants than rs651007 (notably rs411988) with NIMA
	- Weak genomic correlations with ABI at rest and ABI after exercise	
		- No nominally significant associations with all NIMA-related SNPs
		- Genomic correlations with presence of plaque and IMT ~0
Hepcidin/ferritin	Nominally significant associations with two NIMA-related SNPs	- No nominally significant associations with four NIMA-related SNPs
		- Genomic correlations with all NIMA ~0
Hepcidin/TS		- No nominally significant associations with all NIMA-related SNPs
		- Genomic correlations with all NIMA ~0
Iron	- Directionally consistent associations of the multi-SNP score with NIMA in women	- No directionally consistent associations of the multi-SNP score with NIMA in men
		- No directionally consistent associations of iron-related SNPs with NIMA
		- No nominally significant associations with all NIMA-related SNPs
		- Genomic correlations with all NIMA ~0
TIBC	- Directionally consistent associations of the multi-SNP score with NIMA in women	- No directionally consistent associations of the multi-SNP score with NIMA in men
		- No directionally consistent associations of iron-related SNPs with NIMA
		- No nominally significant associations with all NIMA-related SNPs
		- Genomic correlations with all NIMA ~0
TS	- Directionally consistent associations of the multi-SNP score with NIMA in women	- No directionally consistent associations of the multi-SNP score with NIMA in men
		- No directionally consistent associations of iron-related SNPs with NIMA
		- No nominally significant associations with all NIMA-related SNPs
		- Genomic correlations with all NIMA ~0

*ABI* indicates ankle-brachial index, *IMT* intima media thickness, *NIMA* non-invasive measurement of atherosclerosis, *SNP* single nucleotide polymorphism, *TIBC* total iron-binding capacity, and *TS* transferrin saturation

## Discussion

In this study, we investigated relationships of iron parameters and hepcidin with NIMA in a sample of the general population by performing an MR approach, assessing associations of NIMA-related SNPs with iron parameters and hepcidin, and studying genomic correlations of iron parameters, hepcidin, and hepcidin ratios with NIMA. Overall, the results suggest a potential role for ferritin and hepcidin in atherosclerosis, and indicate a potential causal role of iron status on NIMA in women (Table 5).

The results from the current study partly confirm the results from our previous observational study [15]. In this previous study we did not find associations of the four iron parameters with NIMA, except for serum iron and TS with IMT in women, although the trend over quartiles of serum iron and TS was not completely consistent. In the current study we found an indication for a potential causal effect of iron status as a whole on NIMA in women only, with a nominally significant association for Q4 vs Q1 of the multi-SNP score and IMT, which might corroborate the original ‘iron hypothesis’. In addition, the findings of our current study provide weak evidence that hepcidin, ferritin and the ratio hepcidin/ferritin are causally related to atherosclerosis, which confirms the extended ‘iron hypothesis’ and results of our observational study that indicated that the iron *distribution*, as determined by serum hepcidin and the ratio hepcidin/ferritin, plays a role in the development of atherosclerosis.

We used three different approaches to assess causality of the associations between hepcidin, iron and atherosclerosis. First, the multi-SNP score that we constructed based on iron-related SNPs showed directionally consistent associations with all NIMA in women, but not in men. This score reflects iron status as a whole, as the eight SNPs that were combined in this score associate with all four iron parameters. It indicates that an increased body iron status increases the risk of atherosclerosis in women, thus corroborating with the original ‘iron hypothesis’. From the single SNP analyses, however, we did not obtain evidence for a causal role of the iron parameters on NIMA. For all but one of the 12 SNPs, directions of effect were inconsistent over the four NIMA and/or inconsistent with the hypothesized direction of effect according to the ‘iron hypothesis’. The exception was rs651007, which is uniquely associated with a decrease in ferritin concentration and which showed a decreased risk of atherosclerosis. However, rs411988, which is also uniquely associated with ferritin with a similar effect estimate as for rs651007, showed far weaker or even no associations with the NIMA. One would expect similar results for these two SNPs [43], thus the MR approach provides contradicting evidence for a causal role of ferritin in atherosclerosis. The fact that results of our multi-SNP score analyses indicated a causal effect of iron status on atherosclerosis in women, whereas results of the single SNP analyses did not, might be due to a difference in power. The combination of multiple variants into a score can increase power, as the score will explain a larger proportion of variation in the iron parameters than the single variants [44]. The causal effects of hepcidin on atherosclerosis could not be studied by an MR approach, as there have been no SNPs identified yet that have been validated for association with hepcidin.

Secondly, the reverse approach of studying associations of NIMA-associated top SNPs with the iron parameters, as was previously done for coronary artery disease and IMT [45], failed to provide evidence for a role of ferritin, iron, TIBC and TS in atherosclerosis. We did not observe any significant and consistent associations, thus indicating no intermediate or pleiotropic effects of these SNPs on all four iron parameters. Furthermore, the nearest genes for NIMA-associated top SNPs are currently not known to be involved in iron metabolism. The NIMA-related SNPs did also not associate with hepcidin, but two of them did show nominally significant associations with the ratio hepcidin/ferritin, with consistency in observed and hypothesized directions of effect. This indicates that the ratio hepcidin/ferritin and thus the body iron distribution might be involved in atherosclerosis. Notably, two previous studies reported the SNPs rs12091564 and rs10218795 in the hemochromatosis type 2 gene (*HFE2*; also known as hemojuvelin [HJV]) to be associated with coronary artery disease (CAD) based on a two-marker association test and haplotype analysis [46, 47]. Defects in *HFE2* lead to a form of juvenile hemochromatosis, which is characterized by a severe iron overload (high serum ferritin, high TS) mainly in the parenchyma due to a low hepcidin/ferritin ratio, occurring typically before the age of 30. This finding is thus in contrast to the extended ‘iron hypothesis’ that it is the iron loading in the reticulo-endothelial system as determined by hepcidin that promotes the development of atherosclerosis. It also contradicts the general clinical impression that hereditary hemochromatosis associated with parenchymal iron overload is not associated with increased atherosclerosis [48]. As the *HFE2* gene is also expressed in heart and skeletal muscle, it could also be that the SNPs in *HFE2* do not necessarily associate with CAD via iron.

Third, genomic correlations of the iron parameters with the NIMA were close to zero, thus indicating that there is no overlap in genetic etiology of the traits. Exception was the modest negative genomic correlation of ferritin with ABI at rest and ABI after exercise, indicating that genetic variants that increase ferritin cause a decrease in ABI. However, these genomic correlations were far from statistically significant and ferritin did not show genomic correlations with other NIMA. We observed a negative genomic correlation of hepcidin with ABI at rest and after exercise, although in our previous observational study we did not find hepcidin to be associated with ABI at rest and ABI after exercise, but only the ratio hepcidin/ferritin [15]. However, the genomic correlation of the ratio hepcidin/ferritin with ABI at rest and after exercise in the current study was close to zero. Furthermore, we found a significant association of hepcidin and hepcidin/ferritin with presence of plaque in our previous observational study, but genomic correlations between these variables in the current study were very weak.

Our results on the potential role of hepcidin and the ratio hepcidin/ferritin in atherosclerosis are in agreement with findings on the role of hepcidin in patient populations and *in vitro* and mice studies, as discussed in our previous study [15]. They are in contrast to a mouse study recently published by Kautz *et al.* [49], which indicated that increased macrophage iron as a result of a loss-of-function ferroportin mutation, mimicking the effect of increased hepcidin concentration, does not promote atherosclerosis. However, studies in a similar mouse model showed that pharmacological suppression of hepcidin does reduce atherosclerosis [50], and that hepcidin overexpression did change plaque composition although not plaque size [51]. Kautz *et al.* explain their discrepant finding by suggesting that the effect of macrophage iron on atherosclerosis could be so small that contradictory conclusions might be reached due to differences in study design [49]. They also propose that hepcidin might be increased locally in macrophages and adipocytes in the plaque environment and may promote local macrophage iron accumulation [49].

There are some aspects that hampered our study. First of all, the size of our study population was limited. Studies that investigated the power of MR analyses indicate that several thousands of individuals are actually needed to allow for powerful MR studies [52, 53]. Our limited sample size resulted in imprecise estimates of genomic correlations, suboptimal power of our MR analysis and low power to identify SNP associations with statistical significance. Consequently, our results are of an explorative character in which we focused on directions of observed effect estimates and their consistency with the hypothesized direction of effect. In addition, the limited sample size decreased the power of our gender-stratified MR analyses, which we performed based on our previous gender-specific findings [15]. Secondly, our estimation of genomic correlations provided us with indirect evidence of a potential causal relation between these traits; strong genomic correlations can be the result of pleiotropy and are not evidence for causality per se. Also, weak genomic correlations do not exclude causal relationships, as causality can also be the result of (shared) environmental factors only. In addition, the genomic correlations that we reported were based on measured autosomal and common SNPs only. Furthermore, the multi-SNP score that we made reflects iron status as a whole and does not enable to conclude which one of the iron parameters is actually causally related to atherosclerosis risk. Finally, causal inference based on the MR approach is only valid if three crucial assumptions hold, as described in the Introduction. It was limited by the fact that most of the 12 SNPs that we included in our MR approach influence more than one of the iron parameters. Furthermore, the SNPs rs1800562 in *HFE* and rs855791 in *TMPRSS6* also showed association with the ratios hepcidin/ferritin and hepcidin/TS [24, 25], and with red blood cell traits (*e.g.,* [54, 55]). In addition, four of the loci that were found in the iron status meta-GWAS have been reported as lipid loci by the Global Lipids Genetics Consortium [56], *i.e., HFE*, *NAT2*, *ABO* and *FADS2*. However, performing the MR analyses using TC, LDL, HDL and TGC as covariates did not change our conclusions. Still, the overlap in iron and lipid loci is substantial and is therefore unlikely to be based on chance only. It might indicate causal relationships between iron status and lipid levels and therefore also atherosclerosis, which was confirmed by the associations of the multi-SNP score with NIMA in women in our current study.

## Conclusions

This study is the first to evaluate the ‘iron hypothesis’ from a genetic point of view. Our results suggest that an increased iron status plays a causal role in the development of atherosclerosis in women. Our results are also suggestive for a potential causal role of ferritin and hepcidin in atherosclerosis. We warrant future studies to exploit any new genetic variants that are found to be associated with serum hepcidin in an MR approach. In addition, we emphasize follow-up of the current study in a larger series including multiple study populations to allow for adequately powered MR studies.

## Availability of supporting data

All data underlying our findings are available upon request without any costs. The data are managed by the Nijmegen Biomedical Study (NBS) project team; see www.nijmegenbiomedischestudie.nl for an overview of the data available in this study. We cannot make the data underlying the findings of the current manuscript freely available, as we signed a Data Transfer Agreement in order to receive the data in which it is also stated that we cannot distribute the data to other parties. Current practical coordinator of the NBS is the first author of this manuscript, dr. T.E. Galesloot. Readers can contact her to request the data (Tessel.Galesloot@radboudumc.nl).

## Additional file

Additional file 1:
**Flow chart of the SNP selection.** Twelve SNPs identified in meta-GWAS for iron parameters and included in the Mendelian randomization analysis. SNPs identified by published meta-GWAS for IMT, presence of plaque or ABI. Association of the iron-related SNPs with non-invasive measurements of atherosclerosis adjusted for total cholesterol, low-density lipoprotein cholesterol, high-density lipoprotein cholesterol, and triglycerides. Association of the iron-related SNPs with non-invasive measurements of atherosclerosis stratified by gender. Association of the iron-related SNPs with non-invasive measurements of atherosclerosis adjusted for total cholesterol, low-density lipoprotein cholesterol, high-density lipoprotein cholesterol, and triglycerides, and stratified by gender. Associations of NIMA-related SNPs with hepcidin and iron parameters.

## Abbreviations

ABI: Ankle-brachial index
ABIEX: Ankle-brachial index after exercise
ABIR: Ankle-brachial index at rest
CAD: Coronary artery disease
CI: Confidence interval
CVD: Cardiovascular disease
GRM: Genetic relationship matrix
GWAS: Genome-wide association study
HDL: High-density lipoprotein cholesterol
HFE: Hemochromatosis gene
HWE: Hardy-Weinberg Equilibrium
IMT: Intima media thickness
LDL: Low-density lipoprotein cholesterol
MAF: Minor allele frequency
MR: Mendelian randomization
NBS: Nijmegen Biomedical Study
NIMA: Non-invasive measurements of atherosclerosis
OR: Odds ratio
Q: Quartile
QN: Questionnaire
rG: Genomic correlation
SD: Standard Deviation
SE: Standard Error
SNP: Single nucleotide polymorphism
TC: Total cholesterol
TGC: Triglycerides
TIBC: Total-iron binding capacity
TMPRSS6: Transmembrane serine protease 6 gene
TS: Transferrin saturation

## References

[1] Sullivan JL (1981). Iron and the sex difference in heart disease risk. Lancet..

[2] Lapenna D, Pierdomenico SD, Ciofani G, Ucchino S, Neri M, Giamberardino M (2007). Association of body iron stores with low molecular weight iron and oxidant damage of human atherosclerotic plaques. Free Radic Biol Med..

[3] Kraml PJ, Klein RL, Huang Y, Nareika A, Lopes-Virella MF (2005). Iron loading increases cholesterol accumulation and macrophage scavenger receptor I expression in THP-1 mononuclear phagocytes. Metabolism..

[4] Kiechl S, Willeit J, Egger G, Poewe W, Oberhollenzer F (1997). Body iron stores and the risk of carotid atherosclerosis: prospective results from the Bruneck study. Circulation..

[5] Salonen JT, Tuomainen TP, Salonen R, Lakka TA, Nyyssönen K (1998). Donation of blood is associated with reduced risk of myocardial infarction. The Kuopio Ischaemic Heart Disease Risk Factor Study. Am J Epidemiol.

[6] Meyers DG, Jensen KC, Menitove JE (2002). A historical cohort study of the effect of lowering body iron through blood donation on incident cardiac events. Transfusion..

[7] Meyers DG, Strickland D, Maloley PA, Seburg JK, Wilson JE, McManus BF (1997). Possible association of a reduction in cardiovascular events with blood donation. Heart..

[8] Ascherio A, Rimm EB, Giovannucci E, Willett WC, Stampfer MJ (2001). Blood donations and risk of coronary heart disease in men. Circulation..

[9] Zheng H, Cable R, Spencer B, Votto N, Katz SD (2005). Iron stores and vascular function in voluntary blood donors. Arterioscler Thromb Vasc Biol..

[10] Engberink MF, Geleijnse JM, Durga J, Swinkels DW, de Kort WL, Schouten EG (2008). Blood donation, body iron status and carotid intima-media thickness. Atherosclerosis..

[11] Peffer K, den Heijer M, Holewijn S, de Graaf J, Swinkels DW, Verbeek AL (2012). The effect of frequent whole blood donation on ferritin, hepcidin, and subclinical atherosclerosis. Transfusion..

[12] Grammer TB, Kleber ME, Silbernagel G, Pilz S, Scharnagl H, Tomaschitz A (2014). Hemoglobin, iron metabolism and angiographic coronary artery disease (The Ludwigshafen Risk and Cardiovascular Health Study). Atherosclerosis..

[13] Roy CN, Mak HH, Akpan I, Losyev G, Zurakowski D, Andrews NC (2007). Hepcidin antimicrobial peptide transgenic mice exhibit features of the anemia of inflammation. Blood..

[14] Sullivan JL (2007). Macrophage iron, hepcidin, and atherosclerotic plaque stability. Exp Biol Med..

[15] Galesloot TE, Holewijn S, Kiemeney LA, de Graaf J, Vermeulen SH, Swinkels DW (2014). Serum hepcidin is associated with presence of plaque in postmenopausal women of a general population. Arterioscler Thromb Vasc Biol..

[16] Smith GD, Ebrahim S (2003). 'Mendelian randomization': can genetic epidemiology contribute to understanding environmental determinants of disease?. Int J Epidemiol..

[17] Benyamin B, Ferreira MA, Willemsen G, Gordon S, Middelberg RP, McEvoy BP (2009). Common variants in TMPRSS6 are associated with iron status and erythrocyte volume. Nat Genet..

[18] Benyamin B, McRae AF, Zhu G, Gordon S, Henders AK, Palotie A (2009). Variants in TF and HFE explain approximately 40 % of genetic variation in serum-transferrin levels. Am J Hum Genet..

[19] Tanaka T, Roy CN, Yao W, Matteini A, Semba RD, Arking D (2010). A genome-wide association analysis of serum iron concentrations. Blood..

[20] Pichler I, Minelli C, Sanna S, Tanaka T, Schwienbacher C, Naitza S (2011). Identification of a common variant in the TFR2 gene implicated in the physiological regulation of serum iron levels. Hum Mol Genet..

[21] McLaren CE, Garner CP, Constantine CC, McLachlan S, Vulpe CD, Snively BM (2011). Genome-wide association study identifies genetic loci associated with iron deficiency. PLoS One..

[22] Oexle K, Ried JS, Hicks AA, Tanaka T, Hayward C, Bruegel M (2011). Novel association to the proprotein convertase PCSK7 gene locus revealed by analysing soluble transferrin receptor (sTfR) levels. Hum Mol Genet..

[23] Benyamin B, Esko T, Ried JS, Radhakrishnan A, Vermeulen SH, Traglia M (2014). Novel loci affecting iron homeostasis and their effects in individuals at risk for hemochromatosis. Nat Commun..

[24] Traglia M, Girelli D, Biino G, Campostrini N, Corbella M, Sala C (2011). Association of HFE and TMPRSS6 genetic variants with iron and erythrocyte parameters is only in part dependent on serum hepcidin concentrations. J Med Genet..

[25] Galesloot TE, Geurts-Moespot AJ, den Heijer M, Sweep FC, Fleming RE, Kiemeney LA (2013). Associations of common variants in HFE and TMPRSS6 with iron parameters are independent of serum hepcidin in a general population: a replication study. J Med Genet..

[26] Bis JC, Kavousi M, Franceschini N, Isaacs A, Abecasis GR, Schminke U (2011). Meta-analysis of genome-wide association studies from the CHARGE consortium identifies common variants associated with carotid intima media thickness and plaque. Nat Genet..

[27] Murabito JM, White CC, Kavousi M, Sun YV, Feitosa MF, Nambi V (2012). Association between chromosome 9p21 variants and the ankle-brachial index identified by a meta-analysis of 21 genome-wide association studies. Circ Cardiovasc Genet..

[28] Hoogendoorn EH, Hermus AR, de Vegt F, Ross HA, Verbeek AL, Kiemeney LA (2006). Thyroid function and prevalence of anti-thyroperoxidase antibodies in a population with borderline sufficient iodine intake: influences of age and sex. Clin Chem..

[29] Kiemeney LA, Thorlacius S, Sulem P, Geller F, Aben KK, Stacey SN (2008). Sequence variant on 8q24 confers susceptibility to urinary bladder cancer. Nat Genet..

[30] Galesloot TE, Vermeulen SH, Geurts-Moespot AJ, Klaver SM, Kroot JJ, van Tienoven D (2011). Serum hepcidin: reference ranges and biochemical correlates in the general population. Blood..

[31] Kroot JJ, Laarakkers CM, Geurts-Moespot AJ, Grebenchtchikov N, Pickkers P, van Ede AE (2010). Immunochemical and mass-spectrometry-based serum hepcidin assays for iron metabolism disorders. Clin Chem..

[32] Holewijn S, den Heijer M, Swinkels DW, Stalenhoef AF, de Graaf J (2009). The metabolic syndrome and its traits as risk factors for subclinical atherosclerosis. J Clin Endocrinol Metab..

[33] Howie BN, Donnelly P, Marchini J (2009). A flexible and accurate genotype imputation method for the next generation of genome-wide association studies. PLoS Genet..

[34] Yang J, Lee SH, Goddard ME, Visscher PM (2011). GCTA: a tool for genome-wide complex trait analysis. Am J Hum Genet..

[35] Janss L, de Los CG, Sheehan N, Sorensen D (2012). Inferences from genomic models in stratified populations. Genetics..

[36] Lee SH, Yang J, Goddard ME, Visscher PM, Wray NR (2012). Estimation of pleiotropy between complex diseases using single-nucleotide polymorphism-derived genomic relationships and restricted maximum likelihood. Bioinformatics..

[37] Lee SH, Ripke S, Neale BM, Faraone SV, Purcell SM, Cross-Disorder Group of the Psychiatric Genomics C (2013). Genetic relationship between five psychiatric disorders estimated from genome-wide SNPs. Nat Genet.

[38] Visscher PM, Hemani G, Vinkhuyzen AA, Chen GB, Lee SH, Wray NR (2014). Statistical power to detect genetic (co)variance of complex traits using SNP data in unrelated samples. PLoS Genet..

[39] Yang J, Benyamin B, McEvoy BP, Gordon S, Henders AK, Nyholt DR (2010). Common SNPs explain a large proportion of the heritability for human height. Nat Genet..

[40] Bouwman AC, Valente BD, Janss LL, Bovenhuis H, Rosa GJ (2014). Exploring causal networks of bovine milk fatty acids in a multivariate mixed model context. Genet Sel Evol..

[41] Spiegelhalter DJ, Best NG, Carlin BP, van der Linde A (2002). Bayesian measures of model complexity and fit. J Royal Stat Soc: Ser B (Stat Methodol).

[42] Purcell S, Neale B, Todd-Brown K, Thomas L, Ferreira MA, Bender D (2007). PLINK: a tool set for whole-genome association and population-based linkage analyses. Am J Hum Genet..

[43] Palmer TM, Lawlor DA, Harbord RM, Sheehan NA, Tobias JH, Timpson NJ (2012). Using multiple genetic variants as instrumental variables for modifiable risk factors. Stat Methods Med Res..

[44] Pierce BL, Ahsan H, Vanderweele TJ (2011). Power and instrument strength requirements for Mendelian randomization studies using multiple genetic variants. Int J Epidemiol..

[45] Conde L, Bevan S, Sitzer M, Klopp N, Illig T, Thiery J (2012). Novel associations for coronary artery disease derived from genome wide association studies are not associated with increased carotid intima-media thickness, suggesting they do not act via early atherosclerosis or vessel remodeling. Atherosclerosis..

[46] Slavin TP, Feng T, Schnell A, Zhu X, Elston RC (2011). Two-marker association tests yield new disease associations for coronary artery disease and hypertension. Hum Genet..

[47] Zhu X, Feng T, Li Y, Lu Q, Elston RC (2010). Detecting rare variants for complex traits using family and unrelated data. Genet Epidemiol..

[48] Sullivan JL (2009). Do hemochromatosis mutations protect against iron-mediated atherogenesis?. Circ Cardiovasc Genet..

[49] Kautz L, Gabayan V, Wang X, Wu J, Onwuzurike J, Jung G (2013). Testing the iron hypothesis in a mouse model of atherosclerosis. Cell Rep..

[50] Saeed O, Otsuka F, Polavarapu R, Karmali V, Weiss D, Davis T (2012). Pharmacological suppression of hepcidin increases macrophage cholesterol efflux and reduces foam cell formation and atherosclerosis. Arterioscler Thromb Vasc Biol..

[51] Li JJ, Meng X, Si HP, Zhang C, Lv HX, Zhao YX (2012). Hepcidin destabilizes atherosclerotic plaque via overactivating macrophages after erythrophagocytosis. Arterioscler Thromb Vasc Biol..

[52] Burgess S (2014). Sample size and power calculations in Mendelian randomization with a single instrumental variable and a binary outcome. Int J Epidemiol..

[53] Oexle K, Schormair B, Ried JS, Czamara D, Heim K, Frauscher B (2013). Dilution of candidates: the case of iron-related genes in restless legs syndrome. Eur J Hum Genet..

[54] Soranzo N, Sanna S, Wheeler E, Gieger C, Radke D, Dupuis J (2010). Common variants at 10 genomic loci influence hemoglobin A(1)(C) levels via glycemic and nonglycemic pathways. Diabetes..

[55] Kullo IJ, Ding K, Jouni H, Smith CY, Chute CG (2010). A genome-wide association study of red blood cell traits using the electronic medical record. PLoS One..

[56] Willer CJ, Schmidt EM, Sengupta S, Peloso GM, Gustafsson S, Global Lipids Genetics C (2013). Discovery and refinement of loci associated with lipid levels. Nat Genet..

