# Supplementary material for: Iron and hepcidin as risk factors in atherosclerosis: what do the genes say?
Source: BMC Genet. 2015 Jul 11;16:79. doi: 10.1186/s12863-015-0246-4 (PMC4498499; doi:10.1186/s12863-015-0246-4)
Supplement: Additional file 1: — Flow chart of the SNP selection. Twelve SNPs identified in meta-GWAS for iron parameters and included in the Mendelian randomization analysis. SNPs identified by published meta-GWAS for IMT, presence of plaque or ABI. Association of the iron-related SNPs with non-invasive measurements of atherosclerosis adjusted for total cholesterol, low-density lipoprotein cholesterol, high-density lipoprotein cholesterol, and triglycerides. Association of the iron-related SNPs with non-invasive measurements of atherosclerosis stratified by gender. Association of the iron-related SNPs with non-invasive measurements of atherosclerosis adjusted for total cholesterol, low-density lipoprotein cholesterol, high-density lipoprotein cholesterol, and triglycerides, and stratified by gender. Associations of NIMA-related SNPs with hepcidin and iron parameters. [file 12863_2015_246_MOESM1_ESM.docx]

**Additional file**

**Additional file 1: Figure S1.** Flow chart of the SNP selection.

**
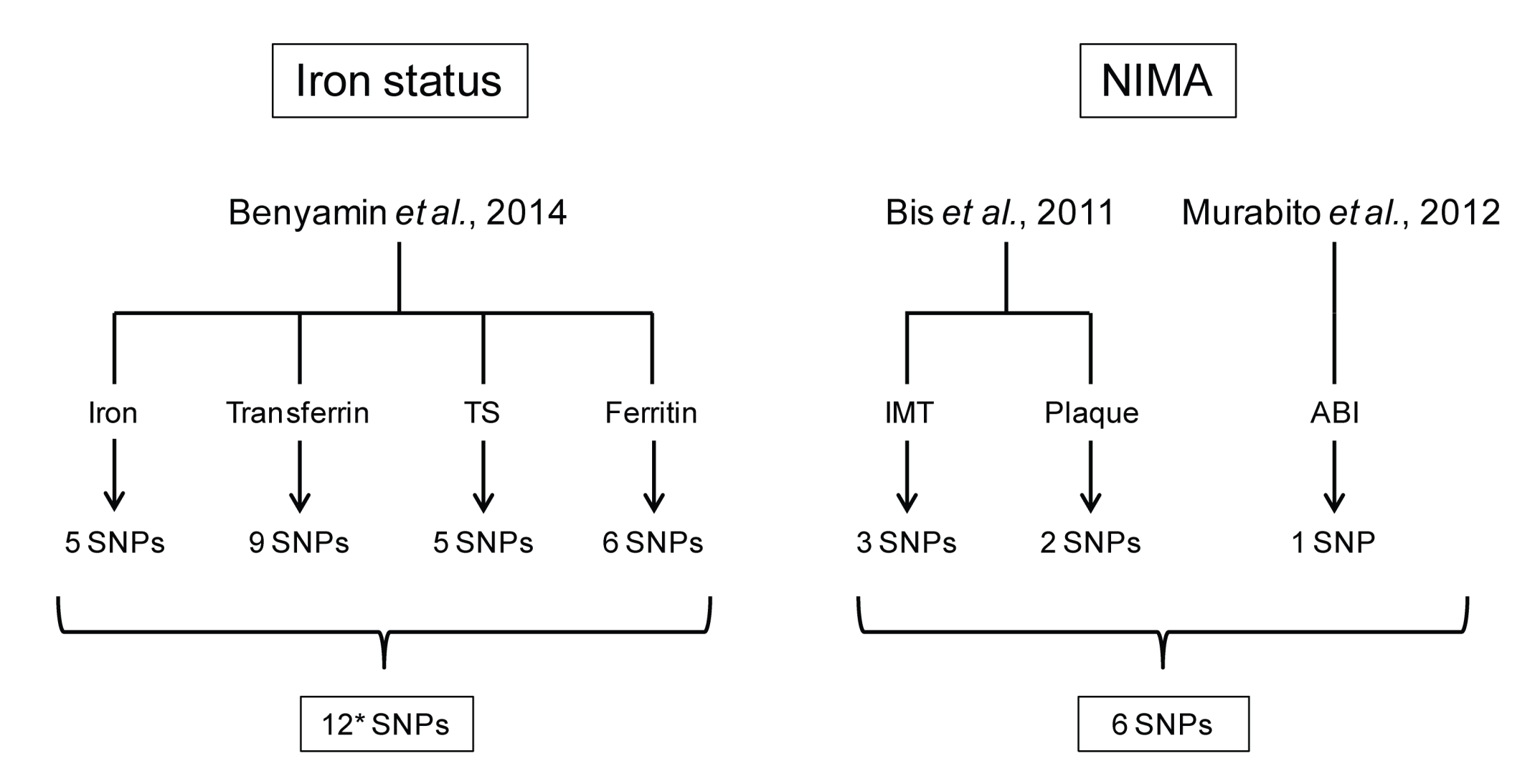
**

ABI indicates ankle-brachial index; IMT, intima media thickness; NIMA, non-invasive measurements of atherosclerosis; SNP, single nucleotide polymorphism; TS, transferrin saturation.

*SNPs add up to 12, because six of the SNPs are associated with more than one iron trait.

**Additional Table S1.** Twelve SNPs identified in meta-GWAS for iron parameters and included in the Mendelian randomization analysis.

|  |  |  |  |  | **Iron** | | | **Transferrin** | | | **TS** | | | **Ferritin (log)** | | |
| --- | --- | --- | --- | --- | --- | --- | --- | --- | --- | --- | --- | --- | --- | --- | --- | --- |
| **SNP** | **Nearest gene(s)** | **Effect allele** | **Freq*** | **HWE p*** | **Beta** | **SE** | **p** | **Beta** | **SE** | **p** | **Beta** | **SE** | **p** | **Beta** | **SE** | **p** |
| rs744653 | *WDR75-SLC40A1* | T | 0.86 | 1.1E-01 | 0.004 | 0.010 | 7.0E-01 | **0.068** | **0.010** | **1.4E-11** | -0.028 | 0.011 | 8.4E-03 | **-0.089** | **0.010** | **8.4E-19** |
| rs8177240 | *TF* | T | 0.34 | 3.7E-01 | **-0.066** | **0.007** | **6.7E-20** | **-0.380** | **0.007** | **<1E-340** | **0.100** | **0.008** | **7.2E-38** | 0.021 | 0.007 | 3.9E-03 |
| rs9990333 | *TFRC* | T | 0.47 | 7.1E-01 | 0.017 | 0.007 | 1.4E-02 | **-0.051** | **0.007** | **2.0E-13** | 0.039 | 0.007 | 7.3E-08 | 0.001 | 0.007 | 8.8E-01 |
| rs1800562 | *HFE* | A | 0.06 | 3.1E-01 | **0.328** | **0.016** | **2.7E-97** | **-0.479** | **0.016** | **8.9E-196** | **0.577** | **0.016** | **2.2E-270** | **0.204** | **0.016** | **1.5E-38** |
| rs1799945 | *HFE* | C | 0.16 | 2.1E-02 | **-0.189** | **0.010** | **1.10E-81** | **0.114** | **0.010** | **9.4E-30** | **-0.231** | **0.010** | **5.1E-109** | **-0.065** | **0.010** | **1.7E-10** |
| rs7385804 | *TFR2* | A | 0.63 | 2.4E-01 | **0.064** | **0.007** | **1.4E-18** | -0.003 | 0.007 | 7.3E-01 | **0.054** | **0.008** | **6.1E-12** | 0.015 | 0.007 | 3.9E-02 |
| rs4921915 | *NAT2* | A | 0.78 | 4.9E-01 | 0.004 | 0.009 | 6.3E-01 | **0.079** | **0.009** | **7.1E-19** | -0.026 | 0.009 | 3.6E-03 | 0.001 | 0.009 | 8.9E-01 |
| rs651007 | *ABO* | T | 0.21 | 5.6E-01 | -0.004 | 0.009 | 6.1E-01 | -0.001 | 0.009 | 9.2E-01 | -0.006 | 0.009 | 5.0E-01 | **-0.050** | **0.009** | **1.3E-08** |
| rs6486121 | *ARNTL* | T | 0.64 | 8.2E-02 | -0.009 | 0.007 | 2.0E-01 | **-0.046** | **0.007** | **3.9E-10** | 0.015 | 0.008 | 4.8E-02 | 0.006 | 0.007 | 4.2E-01 |
| rs174577 | *FADS2* | A | 0.33 | 4.2E-02 | 0.001 | 0.007 | 8.8E-01 | **0.062** | **0.007** | **2.3E-17** | -0.025 | 0.008 | 1.6E-03 | -0.012 | 0.007 | 9.8E-02 |
| rs411988 | *TEX14* | A | 0.58 | 3.3E-01 | -0.002 | 0.007 | 7.7E-01 | 0.014 | 0.007 | 5.2E-02 | -0.012 | 0.007 | 1.2E-01 | **-0.044** | **0.007** | **1.6E-10** |
| rs855791 | *TMPRSS6* | A | 0.54 | 2.7E-01 | **-0.181** | **0.007** | **1.3E-139** | **0.044** | **0.007** | **2.0E-09** | **-0.190** | **0.008** | **6.4E-137** | **-0.055** | **0.007** | **1.4E-14** |

Betas, SE and p-values for iron, transferrin, TS and ferritin based on the findings of the Genetics of Iron Status (GIS) Consortium iron parameter meta-GWAS (discovery and replication combined) (N=48,972; including Nijmegen Biomedical Study) [1]. Genome-wide significant associations (p-value < 5E-08) are indicated in bold. The effect alleles are the same as in the original publication [1].

Freq indicates frequency; HWE, Hardy-Weinberg equilibrium; SE, standard error; SNP, single nucleotide polymorphism; TS, transferrin saturation.

*Effect allele frequency and HWE p-value in Nijmegen Biomedical Study.

**Additional Table S2.** SNPs identified by published meta-GWAS for IMT, presence of plaque or ABI.

| **Trait** | **SNP** | **Nearest gene** | **Effect allele** | **Freq*** | **HWE p*** | **Beta / OR**† | **SE / 95% CI**† | **p** |
| --- | --- | --- | --- | --- | --- | --- | --- | --- |
| IMT | rs11781551 | *ZHX2* | A | 0.46 | 0.17 | -0.0078 | 0.0012 | 2.4E-11 |
|  | rs445925 | *APOC1* | A | 0.11 | 1 | -0.0156 | 0.0028 | 1.7E-08 |
|  | rs6601530 | *PINX1* | G | 0.56 | 0.54 | 0.0078 | 0.0014 | 1.7E-08 |
| Presence of plaque | rs17398575 | *PIK3CG* | A | 0.23 | 0.12 | 1.18 | 1.12; 1.23 | 2.3E-12 |
|  | rs1878406 | *EDNRA* | T | 0.14 | 0.54 | 1.22 | 1.15; 1.29 | 6.9E-12 |
| ABI | rs10757269 | *CDKN2B* | G | 0.46 | 0.60 | -0.0049 | 0.0008 | 2.65E-09 |

Betas, 95% and p-values for SNPs associated with IMT or presence of plaque are based on a meta-analysis of GWAS by Bis *et al.*, 2011 [2]. Betas, 95% and p-values for the SNP associated with ABI are based on a meta-analysis of GWAS by Murabito *et al.*, 2012 [3]. The effect alleles are the same as in the original publications [2,3].

ABI indicates ankle-brachial index; CI, confidence interval; Freq, frequency; HWE, Hardy-Weinberg equilibrium; IMT, intima media thickness; SE, standard error; SNP, single nucleotide polymorphism; TS, transferrin saturation.

*Effect allele frequency and HWE p-value in NBS.

†Beta and SE for SNPs associated with IMT and ABI; OR and 95% CI for SNPs associated with presence of plaque.

**Additional Table S3.** Association of the iron-related SNPs with non-invasive measurements of atherosclerosis adjusted for total cholesterol, low-density lipoprotein cholesterol, high-density lipoprotein cholesterol, and triglycerides.

|  |  | **Presence of plaque** | | |  | **IMT** | | |  | **ABI at rest** | | |  | **ABI after exercise** | | |
| --- | --- | --- | --- | --- | --- | --- | --- | --- | --- | --- | --- | --- | --- | --- | --- | --- |
| **SNP – Tested allele** |  | **H*** | **OR** | **95% CI** |  | **H*** | **Beta** | **95% CI** |  | **H*** | **Beta** | **95% CI** |  | **H*** | **Beta** | **95% CI** |
| Multi-SNP score |  |  |  |  |  |  |  |  |  |  |  |  |  |  |  |  |
| Q1 |  | Ref | Ref | Ref |  | Ref | Ref | Ref |  | Ref | Ref | Ref |  | Ref | Ref | Ref |
| Q2 |  | >1 | 1.17 | 0.72; 1.90 |  | + | -0.015 | -0.039; 0.010 |  | - | 0.002 | -0.023; 0.027 |  | - | -0.015 | -0.039; 0.010 |
| Q3 |  | >1 | 0.88 | 0.53; 1.43 |  | + | -0.001 | -0.026; 0.024 |  | - | 0.005 | -0.020; 0.030 |  | - | -0.001 | -0.026; 0.024 |
| Q4 |  | >1 | 1.12 | 0.68; 1.82 |  | + | 0.005 | -0.020; 0.030 |  | - | 0.001 | -0.024; 0.026 |  | - | 0.005 | -0.020; 0.030 |
| rs744653 – T |  | <1 | 1.15 | 0.80; 1.63 |  | - | -0.004 | -0.022; 0.014 |  | + | -0.012 | -0.030; 0.006 |  | + | -0.013 | -0.046; 0.019 |
| rs8177240 – T† |  | ? | 1.10 | 0.85; 1.44 |  | ? | -0.011 | -0.024; 0.002 |  | ? | 0.013 | -0.001; 0.026 |  | ? | **0.031** | **0.007; 0.056** |
| rs9990333 – T |  | >1 | **1.33** | **1.04; 1.70** |  | + | 0.010 | -0.003; 0.022 |  | - | 0.007 | -0.005; 0.020 |  | - | 0.016 | -0.007; 0.039 |
| rs1800562 – A |  | >1 | 1.11 | 0.66; 1.85 |  | + | -0.008 | -0.034; 0.018 |  | - | -0.008 | -0.034; 0.018 |  | - | -0.007 | -0.055; 0.041 |
| rs1799945 – C |  | <1 | 1.17 | 0.85; 1.62 |  | - | 0.009 | -0.007; 0.025 |  | + | 0.002 | -0.014; 0.019 |  | + | -0.009 | -0.039; 0.021 |
| rs7385804 – A |  | >1 | 0.92 | 0.71; 1.18 |  | + | -0.007 | -0.020; 0.006 |  | - | 0.000 | -0.013; 0.013 |  | - | 0.019 | -0.004; 0.043 |
| rs4921915 – A‡ |  | ? | 1.35 | 0.99; 1.85 |  | ? | -0.011 | -0.027; 0.004 |  | ? | 0.004 | -0.012; 0.019 |  | ? | 0.008 | -0.020; 0.037 |
| rs651007 – T |  | <1 | 0.88 | 0.64; 1.21 |  | - | **-0.016** | **-0.032; 0.000** |  | + | 0.004 | -0.013; 0.020 |  | + | **0.031** | **0.001; 0.060** |
| rs6486121 – T‡ |  | ? | 0.93 | 0.72; 1.20 |  | ? | 0.012 | -0.001; 0.025 |  | ? | -0.003 | -0.016; 0.011 |  | ? | -0.006 | -0.031; 0.018 |
| rs174577 – A‡ |  | ? | 1.14 | 0.88; 1.48 |  | ? | 0.003 | -0.010; 0.016 |  | ? | 0.003 | -0.010; 0.017 |  | ? | 0.014 | -0.010; 0.039 |
| rs411988 – A |  | <1 | 0.91 | 0.72; 1.16 |  | - | -0.001 | -0.014; 0.011 |  | + | 0.004 | -0.008; 0.017 |  | + | -0.009 | -0.032; 0.013 |
| rs855791 – A |  | <1 | 1.21 | 0.94; 1.56 |  | - | -0.007 | -0.020; 0.005 |  | + | 0.005 | -0.007; 0.018 |  | + | 0.010 | -0.014; 0.033 |

Associations were tested using logistic (presence of plaque) and linear regression (IMT and ABI at rest and after exercise). Resulting odds ratios (OR) of logistic models for the multi-SNP score express the change in odds for presence of plaque relative to Q1. Resulting betas of linear models for the multi-SNP score express the change in IMT or ABI using Q1 as a reference, thus Q2 vs Q1, Q3 vs Q1 and Q4 vs Q1. Resulting ORs of logistic regression models for the single SNP analyses express the effect of each extra tested allele on odds for presence of plaque. Resulting betas of linear models for the single SNP analyses express the effect of each extra tested allele on IMT or ABI. Nominally significant associations are indicated in bold.

ABI indicates ankle-brachial index; CI, confidence interval; H, hypothesized effect; IMT, intima media thickness; OR, odds ratio; Ref, reference category; Q, quartile; SNP, single nucleotide polymorphism.

*Hypothesized effect on the NIMA according to the ‘iron hypothesis’ (see Figure 7.1). Presence of plaque, a higher IMT and a lower ABI indicate presence of atherosclerosis.

†This SNP decreases iron and transferrin and increases TS, so the hypothesized effect on atherosclerosis is unknown.

‡These SNPs only show association with transferrin, so the hypothesized effect on atherosclerosis is unknown.

**Additional Table S4.** Association of the iron-related SNPs with non-invasive measurements of atherosclerosis stratified by gender.

|  |  |  | **Presence of plaque** | | |  | **IMT** | | |  | **ABI at rest** | | |  | **ABI after exercise** | | |
| --- | --- | --- | --- | --- | --- | --- | --- | --- | --- | --- | --- | --- | --- | --- | --- | --- | --- |
| **Gender** | **SNP – Tested allele** |  | **H*** | **OR** | **95% CI** |  | **H*** | **Beta** | **95% CI** |  | **H*** | **Beta** | **95% CI** |  | **H*** | **Beta** | **95% CI** |
| Men | Multi-SNP score |  |  |  |  |  |  |  |  |  |  |  |  |  |  |  |  |
|  | Q1 |  | Ref | Ref | Ref |  | Ref | Ref | Ref |  | Ref | Ref | Ref |  | Ref | Ref | Ref |
|  | Q2 |  | >1 | 1.18 | 0.59; 2.36 |  | + | -0.012 | -0.050; 0.027 |  | - | 0.012 | -0.031; 0.055 |  | - | 0.054 | -0.025; 0.133 |
|  | Q3 |  | >1 | 0.56 | 0.28; 1.10 |  | + | -0.016 | -0.054; 0.022 |  | - | 0.014 | -0.029; 0.056 |  | - | 0.064 | -0.014; 0.141 |
|  | Q4 |  | >1 | 0.98 | 0.49; 1.94 |  | + | -0.018 | -0.057; 0.020 |  | - | 0.004 | -0.039; 0.047 |  | - | 0.048 | -0.031; 0.127 |
|  | rs744653 – T |  | <1 | 1.15 | 0.72; 1.85 |  | - | 0.015 | -0.012; 0.042 |  | + | -0.015 | -0.045; 0.015 |  | + | -0.023 | -0.078; 0.032 |
|  | rs8177240 – T† |  | ? | 1.21 | 0.84; 1.76 |  | ? | **-0.022** | **-0.043; -0.001** |  | ? | 0.020 | -0.003; 0.043 |  | ? | **0.046** | **0.004; 0.089** |
|  | rs9990333 – T |  | >1 | 1.07 | 0.75; 1.52 |  | + | 0.006 | -0.014; 0.026 |  | - | 0.013 | -0.009; 0.036 |  | - | 0.037 | -0.003; 0.078 |
|  | rs1800562 – A |  | >1 | 1.04 | 0.48; 2.26 |  | + | -0.006 | -0.050; 0.038 |  | - | -0.024 | -0.073; 0.025 |  | - | -0.005 | -0.096; 0.085 |
|  | rs1799945 – C |  | <1 | **1.64** | **1.04; 2.58** |  | - | **0.026** | **0.001; 0.051** |  | + | -0.001 | -0.029; 0.027 |  | + | -0.019 | -0.071; 0.033 |
|  | rs7385804 – A |  | >1 | 0.72 | 0.51; 1.03 |  | + | -0.016 | -0.036; 0.004 |  | - | 0.011 | -0.011; 0.033 |  | - | 0.039 | -0.001; 0.079 |
|  | rs4921915 – A‡ |  | ? | 1.18 | 0.76; 1.82 |  | ? | -0.027 | -0.051; -0.002 |  | ? | 0.005 | -0.023; 0.032 |  | ? | 0.012 | -0.038; 0.063 |
|  | rs651007 – T |  | <1 | 0.78 | 0.50; 1.22 |  | - | -0.013 | -0.038; 0.011 |  | + | 0.009 | -0.019; 0.037 |  | + | **0.063** | **0.013; 0.114** |
|  | rs6486121 – T‡ |  | ? | 0.91 | 0.64; 1.29 |  | ? | 0.015 | -0.004; 0.035 |  | ? | -0.012 | -0.034; 0.010 |  | ? | -0.019 | -0.059; 0.021 |
|  | rs174577 – A‡ |  | ? | 1.22 | 0.85; 1.73 |  | ? | -0.008 | -0.028; 0.012 |  | ? | 0.008 | -0.014; 0.031 |  | ? | -0.001 | -0.042; 0.040 |
|  | rs411988 – A |  | <1 | 0.93 | 0.66; 1.30 |  | - | -0.008 | -0.027; 0.011 |  | + | 0.004 | -0.017; 0.026 |  | + | -0.018 | -0.057; 0.022 |
|  | rs855791 – A |  | <1 | 0.86 | 0.60; 1.23 |  | - | -0.006 | -0.026; 0.014 |  | + | 0.010 | -0.012; 0.033 |  | + | 0.019 | -0.023; 0.061 |
|  |  |  |  |  |  |  |  |  |  |  |  |  |  |  |  |  |  |
| Women | Multi-SNP score |  |  |  |  |  |  |  |  |  |  |  |  |  |  |  |  |
|  | Q1 |  | Ref | Ref | Ref |  | Ref | Ref | Ref |  | Ref | Ref | Ref |  | Ref | Ref | Ref |
|  | Q2 |  | >1 | 1.10 | 0.53; 2.27 |  | + | -0.014 | -0.047; 0.020 |  | - | 0.005 | -0.022; 0.033 |  | - | 0.033 | -0.017; 0.083 |
|  | Q3 |  | >1 | 1.33 | 0.65; 2.72 |  | + | -0.014 | -0.048; 0.019 |  | - | -0.008 | -0.036; 0.019 |  | - | 0.009 | -0.041; 0.060 |
|  | Q4 |  | >1 | 1.20 | 0.58; 2.47 |  | + | **0.033** | **0.000; 0.067** |  | - | -0.005 | -0.032; 0.023 |  | - | -0.011 | -0.061; 0.039 |
|  | rs744653 – T |  | <1 | 1.07 | 0.63; 1.80 |  | - | **-0.025** | **-0.049; -0.001** |  | + | -0.011 | -0.031; 0.009 |  | + | -0.015 | -0.052; 0.021 |
|  | rs8177240 – T† |  | ? | 1.00 | 0.69; 1.46 |  | ? | -0.004 | -0.021; 0.014 |  | ? | 0.007 | -0.007; 0.021 |  | ? | 0.016 | -0.011; 0.042 |
|  | rs9990333 – T |  | >1 | 1.37 | 0.96; 1.96 |  | + | 0.002 | -0.015; 0.019 |  | - | -0.003 | -0.017; 0.011 |  | - | 0.002 | -0.024; 0.027 |
|  | rs1800562 – A |  | >1 | 1.26 | 0.64; 2.47 |  | + | -0.009 | -0.042; 0.024 |  | - | 0.010 | -0.016; 0.037 |  | - | -0.009 | -0.058; 0.040 |
|  | rs1799945 – C |  | <1 | 0.84 | 0.53; 1.33 |  | - | -0.004 | -0.026; 0.018 |  | + | 0.006 | -0.012; 0.024 |  | + | 0.006 | -0.027; 0.039 |
|  | rs7385804 – A |  | >1 | 1.19 | 0.82; 1.73 |  | + | 0.004 | -0.014; 0.021 |  | - | -0.010 | -0.025; 0.004 |  | - | -0.003 | -0.029; 0.023 |
|  | rs4921915 – A‡ |  | ? | **1.76** | **1.10; 2.82** |  | ? | 0.010 | -0.010; 0.030 |  | ? | -0.001 | -0.017; 0.015 |  | ? | 0.000 | -0.030; 0.030 |
|  | rs651007 – T |  | <1 | 0.94 | 0.60; 1.48 |  | - | **-0.025** | **-0.046; -0.004** |  | + | -0.001 | -0.018; 0.016 |  | + | 0.006 | -0.026; 0.038 |
|  | rs6486121 – T‡ |  | ? | 0.93 | 0.64; 1.37 |  | ? | 0.006 | -0.012; 0.024 |  | ? | 0.004 | -0.011; 0.019 |  | ? | 0.007 | -0.020; 0.035 |
|  | rs174577 – A‡ |  | ? | 0.93 | 0.63; 1.37 |  | ? | 0.006 | -0.012; 0.024 |  | ? | -0.002 | -0.017; 0.013 |  | ? | **0.033** | **0.006; 0.060** |
|  | rs411988 – A |  | <1 | 0.83 | 0.58; 1.18 |  | - | -0.002 | -0.019; 0.015 |  | + | 0.003 | -0.011; 0.016 |  | + | -0.002 | -0.027; 0.023 |
|  | rs855791 – A |  | <1 | **1.79** | **1.24; 2.58** |  | - | -0.010 | -0.027; 0.007 |  | + | 0.001 | -0.012; 0.015 |  | + | 0.002 | -0.023; 0.027 |

Associations were tested using logistic (presence of plaque) and linear regression (IMT and ABI at rest and after exercise). Resulting odds ratios (OR) of logistic models for the multi-SNP score express the change in odds for presence of plaque relative to Q1. Resulting betas of linear models for the multi-SNP score express the change in IMT or ABI using Q1 as a reference, thus Q2 vs Q1, Q3 vs Q1 and Q4 vs Q1. Resulting ORs of logistic regression models for the single SNP analyses express the effect of each extra tested allele on odds for presence of plaque. Resulting betas of linear models for the single SNP analyses express the effect of each extra tested allele on IMT or ABI. Nominally significant associations are indicated in bold.

ABI indicates ankle-brachial index; CI, confidence interval; H, hypothesized effect; IMT, intima media thickness; OR, odds ratio; Ref, reference category; Q, quartile; SNP, single nucleotide polymorphism.

*Hypothesized effect on the NIMA according to the ‘iron hypothesis’ (see Figure 7.1). Presence of plaque, a higher IMT and a lower ABI indicate presence of atherosclerosis.

†This SNP decreases iron and transferrin and increases TS, so the hypothesized effect on atherosclerosis is unknown.

‡These SNPs only show association with transferrin, so the hypothesized effect on atherosclerosis is unknown.

**Additional Table S5.** Association of the iron-related SNPs with non-invasive measurements of atherosclerosis adjusted for total cholesterol, low-density lipoprotein cholesterol, high-density lipoprotein cholesterol, and triglycerides, and stratified by gender.

|  |  |  | **Presence of plaque** | | |  | **IMT** | | |  | **ABI at rest** | | |  | **ABI after exercise** | | |
| --- | --- | --- | --- | --- | --- | --- | --- | --- | --- | --- | --- | --- | --- | --- | --- | --- | --- |
| **Gender** | **SNP – Tested allele** |  | **H*** | **OR** | **95% CI** |  | **H*** | **Beta** | **95% CI** |  | **H*** | **Beta** | **95% CI** |  | **H*** | **Beta** | **95% CI** |
| Men | Multi-SNP score |  |  |  |  |  |  |  |  |  |  |  |  |  |  |  |  |
|  | Q1 |  | Ref | Ref | Ref |  | Ref | Ref | Ref |  | Ref | Ref | Ref |  | Ref | Ref | Ref |
|  | Q2 |  | >1 | 1.15 | 0.56; 2.32 |  | + | -0.011 | -0.048; 0.025 |  | - | 0.011 | -0.032; 0.054 |  | - | 0.052 | -0.026; 0.130 |
|  | Q3 |  | >1 | 0.56 | 0.28; 1.12 |  | + | -0.010 | -0.046; 0.025 |  | - | 0.010 | -0.033; 0.052 |  | - | 0.052 | -0.025; 0.128 |
|  | Q4 |  | >1 | 0.99 | 0.49; 2.01 |  | + | -0.024 | -0.061; 0.013 |  | - | -0.001 | -0.045; 0.042 |  | - | 0.033 | -0.045; 0.112 |
|  | rs744653 – T |  | <1 | 1.13 | 0.69; 1.85 |  | - | 0.013 | -0.012; 0.039 |  | + | -0.009 | -0.039; 0.021 |  | + | -0.008 | -0.063; 0.047 |
|  | rs8177240 – T† |  | ? | 1.20 | 0.82; 1.76 |  | ? | **-0.020** | **-0.040; 0.000** |  | ? | 0.016 | -0.008; 0.039 |  | ? | **0.044** | **0.002; 0.087** |
|  | rs9990333 – T |  | >1 | 1.06 | 0.73; 1.53 |  | + | 0.006 | -0.014; 0.025 |  | - | 0.013 | -0.009; 0.035 |  | - | 0.035 | -0.005; 0.076 |
|  | rs1800562 – A |  | >1 | 1.04 | 0.47; 2.32 |  | + | -0.007 | -0.049; 0.035 |  | - | -0.020 | -0.068; 0.029 |  | - | 0.003 | -0.086; 0.092 |
|  | rs1799945 – C |  | <1 | **1.60** | **1.00; 2.57** |  | - | 0.020 | -0.004; 0.044 |  | + | 0.001 | -0.027; 0.029 |  | + | -0.016 | -0.067; 0.035 |
|  | rs7385804 – A |  | >1 | 0.72 | 0.50; 1.03 |  | + | -0.016 | -0.035; 0.003 |  | - | 0.011 | -0.011; 0.033 |  | - | 0.038 | -0.002; 0.077 |
|  | rs4921915 – A‡ |  | ? | 1.17 | 0.74; 1.83 |  | ? | **-0.024** | **-0.047; 0.000** |  | ? | 0.005 | -0.023; 0.032 |  | ? | 0.013 | -0.037; 0.063 |
|  | rs651007 – T |  | <1 | 0.82 | 0.52; 1.29 |  | - | -0.009 | -0.033; 0.014 |  | + | 0.009 | -0.018; 0.037 |  | + | **0.061** | **0.011; 0.111** |
|  | rs6486121 – T‡ |  | ? | 0.89 | 0.62; 1.28 |  | ? | 0.016 | -0.003; 0.035 |  | ? | -0.009 | -0.031; 0.013 |  | ? | -0.016 | -0.056; 0.024 |
|  | rs174577 – A‡ |  | ? | 1.26 | 0.88; 1.82 |  | ? | -0.009 | -0.028; 0.010 |  | ? | 0.009 | -0.013; 0.031 |  | ? | 0.001 | -0.039; 0.042 |
|  | rs411988 – A |  | <1 | 0.91 | 0.64; 1.30 |  | - | -0.005 | -0.023; 0.014 |  | + | 0.003 | -0.019; 0.024 |  | + | -0.019 | -0.057; 0.020 |
|  | rs855791 – A |  | <1 | 0.88 | 0.60; 1.27 |  | - | -0.001 | -0.020; 0.019 |  | + | 0.011 | -0.012; 0.034 |  | + | 0.020 | -0.021; 0.061 |
|  |  |  |  |  |  |  |  |  |  |  |  |  |  |  |  |  |  |
| Women | Multi-SNP score |  |  |  |  |  |  |  |  |  |  |  |  |  |  |  |  |
|  | Q1 |  | Ref | Ref | Ref |  | Ref | Ref | Ref |  | Ref | Ref | Ref |  | Ref | Ref | Ref |
|  | Q2 |  | >1 | 1.25 | 0.60; 2.62 |  | + | -0.005 | -0.037; 0.028 |  | - | 0.006 | -0.022; 0.033 |  | - | 0.036 | -0.014; 0.087 |
|  | Q3 |  | >1 | 1.53 | 0.74; 3.18 |  | + | -0.003 | -0.035; 0.030 |  | - | -0.007 | -0.035; 0.021 |  | - | 0.010 | -0.041; 0.061 |
|  | Q4 |  | >1 | 1.31 | 0.63; 2.74 |  | + | **0.037** | **0.005; 0.069** |  | - | -0.006 | -0.033; 0.022 |  | - | -0.005 | -0.056; 0.045 |
|  | rs744653 – T |  | <1 | 1.12 | 0.65; 1.90 |  | - | -0.022 | -0.046; 0.002 |  | + | -0.013 | -0.033; 0.007 |  | + | -0.014 | -0.051; 0.023 |
|  | rs8177240 – T† |  | ? | 0.93 | 0.67; 1.44 |  | ? | -0.006 | -0.023; 0.012 |  | ? | 0.007 | -0.007; 0.021 |  | ? | 0.016 | -0.010; 0.043 |
|  | rs9990333 – T |  | >1 | 1.40 | 0.98; 2.02 |  | + | 0.004 | -0.012; 0.020 |  | - | -0.003 | -0.017; 0.010 |  | - | 0.001 | -0.024; 0.026 |
|  | rs1800562 – A |  | >1 | 1.30 | 0.65; 2.58 |  | + | -0.003 | -0.035; 0.029 |  | - | 0.007 | -0.020; 0.034 |  | - | -0.008 | -0.057; 0.042 |
|  | rs1799945 – C |  | <1 | 0.81 | 0.51; 1.29 |  | - | -0.003 | -0.025; 0.018 |  | + | 0.007 | -0.011; 0.025 |  | + | 0.003 | -0.030; 0.036 |
|  | rs7385804 – A |  | >1 | 1.21 | 0.83; 1.78 |  | + | 0.004 | -0.013; 0.021 |  | - | -0.011 | -0.026; 0.003 |  | - | -0.001 | -0.027; 0.025 |
|  | rs4921915 – A‡ |  | ? | **1.74** | **1.08; 2.81** |  | ? | 0.002 | -0.018; 0.022 |  | ? | 0.002 | -0.015; 0.018 |  | ? | -0.001 | -0.032; 0.030 |
|  | rs651007 – T |  | <1 | 0.94 | 0.59; 1.48 |  | - | -0.022 | **-0.043; -0.002** |  | + | -0.001 | -0.018; 0.017 |  | + | 0.004 | -0.028; 0.036 |
|  | rs6486121 – T‡ |  | ? | 0.91 | 0.62; 1.35 |  | ? | 0.006 | -0.011; 0.024 |  | ? | 0.005 | -0.010; 0.019 |  | ? | 0.006 | -0.022; 0.033 |
|  | rs174577 – A‡ |  | ? | 0.91 | 0.61; 1.35 |  | ? | 0.009 | -0.009; 0.026 |  | ? | -0.004 | -0.019; 0.011 |  | ? | 0.033 | **0.006; 0.061** |
|  | rs411988 – A |  | <1 | 0.83 | 0.58; 1.19 |  | - | -0.002 | -0.018; 0.014 |  | + | 0.003 | -0.011; 0.016 |  | + | -0.002 | -0.027; 0.023 |
|  | rs855791 – A |  | <1 | **1.75** | **1.20; 2.53** |  | - | -0.013 | -0.030; 0.003 |  | + | 0.001 | -0.013; 0.015 |  | + | 0.001 | -0.024; 0.026 |

Associations were tested using logistic (presence of plaque) and linear regression (IMT and ABI at rest and after exercise). Resulting odds ratios (OR) of logistic models for the multi-SNP score express the change in odds for presence of plaque relative to Q1. Resulting betas of linear models for the multi-SNP score express the change in IMT or ABI using Q1 as a reference, thus Q2 vs Q1, Q3 vs Q1 and Q4 vs Q1. Resulting ORs of logistic regression models for the single SNP analyses express the effect of each extra tested allele on odds for presence of plaque. Resulting betas of linear models for the single SNP analyses express the effect of each extra tested allele on IMT or ABI. Nominally significant associations are indicated in bold.

ABI indicates ankle-brachial index; CI, confidence interval; H, hypothesized effect; IMT, intima media thickness; OR, odds ratio; Ref, reference category; Q, quartile; SNP, single nucleotide polymorphism.

*Hypothesized effect on the NIMA according to the ‘iron hypothesis’ (see Figure 1). Presence of plaque, a higher IMT and a lower ABI indicate presence of atherosclerosis.

†This SNP decreases iron and transferrin and increases TS, so the hypothesized effect on atherosclerosis is unknown.

‡These SNPs only show association with transferrin, so the hypothesized effect on atherosclerosis is unknown.

| **Additional Table S6.** Associations of NIMA-related SNPs with hepcidin and iron parameters. | | | | | | | | | |  |  |  |
| --- | --- | --- | --- | --- | --- | --- | --- | --- | --- | --- | --- | --- |
| **Source** | **Trait** | **SNP** | **Nearest gene** | **Effect (beta or OR) [SE or 95% CI]** | **P-value** | **Trait** | **Tested allele** | **H*** | **Effect** | **SE** | **P** | **N** |
| Bis *et al.*, 2011 | Carotid IMT | rs11781551-A | *ZHX2* | -0.0078 [0.0012] | 2.4E-11 | hepcidin | A | - | -0.03 | 0.03 | 0.33 | 1800 |
|  |  |  |  |  |  | **hepcidin/ferritin** | **A** | **-** | **-0.06** | **0.03** | **0.03** | **1794** |
|  |  |  |  |  |  | hepcidin/TS | A | - | -0.02 | 0.03 | 0.46 | 1780 |
|  |  |  |  |  |  | iron | A | - | 0.02 | 0.03 | 0.60 | 1790 |
|  |  |  |  |  |  | TIBC | A | + | -0.02 | 0.03 | 0.59 | 1787 |
|  |  |  |  |  |  | TS | A | - | 0.04 | 0.03 | 0.29 | 1786 |
|  |  |  |  |  |  | ferritin | A | - | 0.00 | 0.03 | 1.00 | 1809 |
|  | Carotid IMT | rs445925-A | *APOC1* | -0.0156 [0.0028] | 1.7E-08 | hepcidin | G | + | 0.02 | 0.06 | 0.69 | 1800 |
|  |  |  |  |  |  | hepcidin/ferritin | G | + | 0.11 | 0.06 | 0.06 | 1794 |
|  |  |  |  |  |  | hepcidin/TS | G | + | 0.02 | 0.06 | 0.78 | 1780 |
|  |  |  |  |  |  | iron | G | + | -0.02 | 0.06 | 0.78 | 1790 |
|  |  |  |  |  |  | TIBC | G | - | 0.04 | 0.06 | 0.46 | 1787 |
|  |  |  |  |  |  | TS | G | + | -0.05 | 0.06 | 0.37 | 1786 |
|  |  |  |  |  |  | ferritin | G | + | 0.03 | 0.06 | 0.58 | 1809 |
|  | Carotid IMT | rs6601530-G | *PINX1* | 0.0078 [0.0014] | 1.7E-08 | hepcidin | G | + | -0.02 | 0.03 | 0.50 | 1800 |
|  |  |  |  |  |  | hepcidin/ferritin | G | + | -0.01 | 0.03 | 0.77 | 1794 |
|  |  |  |  |  |  | hepcidin/TS | G | + | -0.02 | 0.03 | 0.48 | 1780 |
|  |  |  |  |  |  | iron | G | + | 0.02 | 0.03 | 0.66 | 1790 |
|  |  |  |  |  |  | TIBC | G | - | 0.03 | 0.03 | 0.43 | 1787 |
|  |  |  |  |  |  | TS | G | + | 0.02 | 0.03 | 0.63 | 1786 |
|  |  |  |  |  |  | ferritin | G | + | 0.02 | 0.03 | 0.64 | 1809 |
|  | Presence of plaque | rs17398575-A | *PIK3CG* | 1.18 [1.12-1.23] | 2.3E-12 | hepcidin | A | + | 0.03 | 0.04 | 0.52 | 1800 |
|  |  |  |  |  |  | hepcidin/ferritin | A | + | 0.03 | 0.04 | 0.47 | 1794 |
|  |  |  |  |  |  | hepcidin/TS | A | + | 0.01 | 0.04 | 0.71 | 1780 |
|  |  |  |  |  |  | iron | A | + | -0.01 | 0.04 | 0.80 | 1790 |
|  |  |  |  |  |  | TIBC | A | - | -0.01 | 0.04 | 0.85 | 1787 |
|  |  |  |  |  |  | TS | A | + | 0.00 | 0.04 | 0.92 | 1786 |
|  |  |  |  |  |  | ferritin | A | + | 0.00 | 0.04 | 0.99 | 1809 |

| **Additional Table S6. (continued)** | | |  |  |  |  |  |  |  |  |  |  |
| --- | --- | --- | --- | --- | --- | --- | --- | --- | --- | --- | --- | --- |
| **Source** | **Trait** | **SNP** | **Nearest gene** | **Effect (beta or OR) [SE or 95% CI]** | **P-value** | **Trait** | **Tested allele** | **H*** | **Effect** | **SE** | **P** | **N** |
|  | Presence of plaque | rs1878406-T | *EDNRA* | 1.22 [1.15-1.29] | 6.9E-12 | hepcidin | T | + | 0.03 | 0.05 | 0.60 | 1800 |
|  |  |  |  |  |  | hepcidin/ferritin | T | + | 0.03 | 0.05 | 0.58 | 1794 |
|  |  |  |  |  |  | hepcidin/TS | T | + | 0.03 | 0.05 | 0.58 | 1780 |
|  |  |  |  |  |  | iron | T | + | 0.08 | 0.05 | 0.09 | 1790 |
|  |  |  |  |  |  | TIBC | T | - | 0.01 | 0.05 | 0.91 | 1787 |
|  |  |  |  |  |  | TS | T | + | 0.05 | 0.05 | 0.35 | 1786 |
|  |  |  |  |  |  | ferritin | T | + | 0.01 | 0.05 | 0.88 | 1809 |
| Murabito *et al.*, 2012 | ABI | rs10757269-G | *CDKN2B* | -0.0049 [0.0008] | 2.7E-09 | hepcidin | G | + | 0.01 | 0.03 | 0.70 | 1800 |
|  |  |  |  |  |  | **hepcidin/ferritin** | **G** | **+** | **0.07** | **0.03** | **0.03** | **1794** |
|  |  |  |  |  |  | hepcidin/TS | G | + | 0.01 | 0.03 | 0.67 | 1780 |
|  |  |  |  |  |  | iron | G | + | 0.02 | 0.03 | 0.56 | 1790 |
|  |  |  |  |  |  | TIBC | G | - | 0.02 | 0.03 | 0.81 | 1787 |
|  |  |  |  |  |  | TS | G | + | 0.03 | 0.03 | 0.46 | 1786 |
|  |  |  |  |  |  | ferritin | G | + | 0.05 | 0.03 | 0.15 | 1809 |

Associations were tested using linear regression. Resulting betas express the effect of each extra tested allele standardized residuals of hepcidin, hepcidin ratios and the iron parameters. Nominally significant associations are indicated in bold.

ABI indicates ankle-brachial index; CI, confidence interval; H, hypothesized effect; IMT, intima media thickness; OR, odds ratio; SE, standard error; SNP, single nucleotide polymorphism.

*Hypothesized effect on hepcidin, hepcidin ratios and the iron parameters according to the ‘iron hypothesis’. Presence of plaque, a higher IMT and a lower ABI indicate presence of atherosclerosis.

**References**

1 Benyamin B, Ferreira MA, Willemsen G, Gordon S, Middelberg RP, McEvoy BP, et al. Common variants in TMPRSS6 are associated with iron status and erythrocyte volume. Nat Genet. 2009;41:1173-5.

2 Bis JC, Kavousi M, Franceschini N, Isaacs A, Abecasis GR, Schminke U, et al. Meta-analysis of genome-wide association studies from the CHARGE consortium identifies common variants associated with carotid intima media thickness and plaque. Nat Genet. 2011;43:940-7.

3 Murabito JM, White CC, Kavousi M, Sun YV, Feitosa MF, Nambi V, et al. Association between chromosome 9p21 variants and the ankle-brachial index identified by a meta-analysis of 21 genome-wide association studies. Circ Cardiovasc Genet. 2012;5:100-12.
